# *Coeloglossum viride* Var. *Bracteatum* Extract Attenuates MPTP-Induced Neurotoxicity *in vivo* by Restoring BDNF-TrkB and FGF2-Akt Signaling Axis and Inhibiting RIP1-Driven Inflammation

**DOI:** 10.3389/fphar.2022.903235

**Published:** 2022-04-28

**Authors:** Xiu-Yuan Lang, Yang Hu, Jin-Peng Bai, Jun Wang, Xiao-Yan Qin, Rongfeng Lan

**Affiliations:** ^1^ Key Laboratory of Ecology and Environment in Minority Areas National Ethnic Affairs Commission, Center on Translational Neuroscience, College of Life and Environmental Sciences, Minzu University of China, Beijing, China; ^2^ Department of Cell Biology and Medical Genetics, School of Basic Medical Sciences, Shenzhen University Health Science Center, Shenzhen, China

**Keywords:** BDNF, LC-3, Parkinson’s disease, RIP1, TBK1

## Abstract

The tuber of *Coeloglossum viride* var. *bracteatum* is a Tibetan medicine that has been used for generations as a tonic for Yang and Qi, tranquilizing, to enhance intelligence and to promote longevity. We have previously characterized the constituents of *Coeloglossum viride* var. *bracteatum* extract (CE) and investigated its anti-Alzheimer’s disease (AD) effect in mice models. However, the exact role of CE in Parkinson’s disease (PD), especially the neurotrophic and inflammatory pathways regulated by CE, remains unknown. In this study, we investigated the anti-PD effects of CE in an MPTP-induced acute mouse model and its underlying mechanisms, focusing on BDNF, FGF2 and their mediated signaling pathways and RIP1-driven inflammatory signaling axis. Pole test and traction test were performed for behavioral analysis. RT-PCR, IHC and Western blotting were performed to assay the mRNA, tissues, and protein, respectively. We found that CE improved dyskinesia in MPTP-intoxicated mice, which was confirmed by the pole test and traction test. Also, oxidative stress and astrocyte activation and inflammation were alleviated. MPTP-intoxication disrupted the levels of BDNF, FGF2 and their mediated signaling pathways, triggered elevation of pro-inflammatory factors such as TNF-α, IL-1β, and IL-6, and activated RIP1-driven inflammatory axis. However, CE restored the levels of BDNF, FGF2 and TrkB/Akt signaling pathways while inhibiting the RIP1-driven inflammatory signaling axis, thereby inhibiting apoptosis, preventing loss of nigrostriatal neurons, and maintaining cellular homeostasis. Thus, CE is a promising agent for the treatment of PD.

## Introduction

The tuber of *Coeloglossum viride* var. *bracteatum* is a traditional Tibetan medicine used to strengthen the Yang and Qi, to treat impotence and bronchial asthma (Shang et al., 2017). It has long been used as a tonic for cough, kidney asthenia and dyspnea in Asian countries such as China and Nepal, and is documented in the classical Tibetan medical classics “Si Bu Yi Dian” and “Jing Zhu Ben Cao” (Udo-Gendaan-Gompo, 1983; Dilma-Tenzin-Phuntsok, 2012). The preparations using *Coeloglossum viride* var. *bracteatum* extract (CE) as the main ingredient, such as “Shi Wei Shou Shen San”, “Fu Fang Shou Shen Wan”, have been approved by the State Medical Products Administration of China (https://www.nmpa.gov.cn) and are listed in the Chinese Pharmacopeia. We have previously identified the composition of CE and demonstrated its neuroprotective effects in AlCl_3_/Gal-induced cognitive impairment or 5xFAD Alzheimer’s disease mouse models (Zhong et al., 2019; Cai et al., 2021; Li et al., 2021). CE exerts a large number of antioxidant, anti-aging and anti-inflammatory properties and has no auto-toxicity, thus a potential drug for the treatment of neurodegenerative diseases (Shang et al., 2017). CE is mainly composed of a large number of glycosides, such as Militarine, Dactylorhin A, Dactylorhin B, Loroglossin, and Coelovirin A and so on. Increasing evidence suggests that the mechanism of CE activity depends on attenuating oxidative stress and inflammation and enhancing the activity of neurotrophic factors, including BDNF, FGF2, etc. and their regulated downstream signaling pathways, such as PI3K-Akt/Bcl-2, and Erk1/2 (Shang et al., 2017; Li et al., 2022). In contrast, glial cell activation and the typical inflammatory process they mediate and the subsequent release of pro-inflammatory factors such as TNF-α, IL-1β, and IL-6 are inhibited by CE. As a result, neuronal apoptosis is inhibited and the cellular homeostasis of the central nervous system (CNS) is maintained. Indeed, we have previously investigated the anti-PD and antidepressant effects of 2,3,5,4′-tetrahydoxystilbene-2-O-β-D-glucoside (THSG) in cultured cells and MPTP-induced PD mouse models as well as in mice with chronic stress-induced depression (Yu et al., 2019; Li et al., 2020). THSG is a potent agent that increases activity of FGF2 and BDNF levels and their downstream signaling pathways in the CNS. THSG inhibits apoptosis and promotes cell survival, thereby improving dyskenesia in PD mouse models.

MPTP (1-methyl-4-phenyl-1, 2, 3, 6-tetrahydropyridine) is non-toxic and permeable and accumulates in the CNS after crossing the blood-brain barrier, especially in astrocytes, where it is metabolized to toxic 1-methyl-4-phenylpyridinium (MPP+). MPP + then destroys dopaminergic neurons in the substantia nigra and striatum, leading to permanent PD-like symptoms (Dauer and Przedborski, 2003). Indeed, the loss of mesencephalic dopaminergic neurons is the most remarkable feature of PD pathogenesis and of the MPTP-induced murine models. Tyrosine hydroxylase (TH), the rate-limiting enzyme of catecholamine biosynthesis (Dauer and Przedborski, 2003; Battaglia et al., 2006), directly regulates levodopa biosynthesis, especially in dopaminergic neurons, and thus it can be used as a marker to monitor nigrostriatal damage. BDNF is a neurtrophic factor of dopaminergic neurons that increases its survival (Hyman et al., 1991). Indeed, one hypothesis proposes that deficiencies of neurotrophins and other regulatory factors lead to neuronal atrophy and apoptosis, and that restoring the expression of these factors and their downstream signaling axes improves neuronal integrity and plasticity (Martinowich et al., 2007; Chen et al., 2016; Zhong et al., 2019). PI3K/Akt-Bcl-2 is the most prevalent signaling axis that maintains cell survival and is actually required for neuronal survival (Long et al., 2021). BDNF acts as an upstream trigger to activate Akt signaling and initiate signaling cascades by binding to the neurotrophic tyrosine kinase receptor TrkB. Thus, BDNF and its mediated signaling is a promising target for neurodegenerative diseases and drug development.

In this study, we investigate the anti-PD effects of CE in an MPTP-induced mouse model. The levels of BDNF, FGF2 and their associated signaling pathways, oxidative stress and glial cell activation-mediated inflammation and RIP1-driven inflammatory pathways were detected to verify the activity of CE as well as the underlying mechanisms.

## Materials and Methods

### Animals

C57BL/6 mice (8–10 weeks, 20 g) were provided by Beijing Vital River Laboratory Animal Technology Co. Ltd. under license No. SCXK-2016-2002. Mice were provided with food and water ad libitum and maintained on a 12-h light/dark cycle. All procedures were performed in accordance with the National Institutes of Health Laboratory Animal Care and Use Guidelines (NIH Publication No. 80-23) and approved by Animal Care and Use Committee of Minzu University of China.

A total of 60 mice were randomly divided into six groups (*n* = 10) of Control, MPTP, MPTP +125 mg/kg Madopar, MPTP +5 mg/kg CE, MPTP +10 mg/kg CE, and MPTP +20 mg/kg CE. The acute mouse PD model was established as described (Jackson-Lewis and Przedborski, 2007). Briefly, MPTP (20 mg/kg) was administrated subcutaneously to the animals four times in 1 day (day 8). From day 1 to day 21, mice in the MPTP + CE and MPTP + Madopar groups received daily oral CE or Madopar, while mice in the control group received equal amounts of saline. Behavioral procedures were performed on days 7, 9 and 21. Finally, mice were anesthetized with 5% chloral hydrate (0.1 ml/g), perfused and dissected in the substantia nigra and striatum for further experiments.

### CE and Chemicals

*Coeloglossum viride* var. *bracteatum* was identified, collected, and processed as previously described (Huang et al., 2004; Shang et al., 2017; Li et al., 2022). In this work, CE was prepared and its chemical composition was characterized according to previous methods (Cai et al., 2021; Li et al., 2021). In brief, the dried tubers of *Coeloglossum viride* var. *bracteatum* was extracted with 70% ethanol at reflux, then suspended in water and partitioned successively with petroleum ether, ethyl acetate, and n-butanol. The n-butanol extract was furthered purified by passing through a HP-20 macroporous resin. The final product used in this study was characterized by a hydrosphere C18 column for the main components of CE, including Dactylorhin A, Dactylorhin B, Loroglossin, and Militarine (Cai et al., 2021). Madopar is a clinical anti-PD drug containing two active ingredients, levodopa (#S2176, Selleck) and benserazide (#S2453, Selleck) in a ratio of 4:1. MPTP was purchased from Sigma-Aldrich (#M0896).

### Behavior Procedures

The Pole test was used to examine the locomotion and motor coordination in PD mice (Matsuura et al., 1997). A 25-mm diameter cork ball was secured to the top of a wooden pole (height 50 cm and diameter 1 cm) with gauze to prevent slippage. Mice were placed face up on top of the cork ball and tested for the time required to descend from the cork ball to the middle of the wooden pole and the time to descend from the middle to the ground. Trials completed within 3 s were scored three points; 3–6 s, two points; > 6 s, one point. Three trails were performed for each animal to obtain an average.

The traction test was used to measure muscle strength and equilibrium (Cryan et al., 2005). Mice are suspended by their front paws from a horizontal rope approximately 40–50 cm above the table to provide sufficient time and space for the animal to land. Duration time was recorded and scored according to the following criteria: 0–4 s, 0 point; 5–9 s, one point; 10–14 s, two points; 15–19 s, three points; 20–24 s, four points; 25–29 s, five points; and >30 s, six points.

### Real-Time PCR

Total RNA was purified from substantia nigra using TRIzol reagent (#15596018, Ambion). First strand cDNAs were yielded from 2.0 μg RNA using EasyQuick RT MasterMix Kit (#CW 2019M, Cowin Bio.) and then diluted 1:10 in nuclease-free water for a 20 μl reaction. Quantitative PCR were performed in a Light Cycler 96 Real-Time PCR System (Roche) using 2x RealStar Green Fast Mixture (#A301-05, GenStar) containing primers for TNF-α, IL-1β, IL-6 and IL-10, respectively, for 20 μl reactions. Three replicates of each dilution were performed and five samples were examined.

### Oxidative Stress Assay

The substantia nigra was isolated and resolved (∼0.3 g) into 1.5 ml of distilled water at 4°C, and homogenates in a grinder (#SCENTZ-48, Ningbo Scientz Biotechnology Co., Ltd.) for 2 min, followed by centrifugation at 3,000 rpm for 10 min collection of the supernatant. Protein concentrations were determined by BCA assay (#P0011, Beyotime Biotechnology).

Total antioxidant capacity (T-AOC) (#A015-1-2), enzymatic activity of superoxide dismutase (T-SOD) (#A001-3-2), and the contents of malondialdehyde (MDA) (#A003-1-2) in substantia nigra were determined by spectrophotometer, and the kits were purchased from Nanjing Jiancheng Biological Engineering Research Institute, according to the manufacturer’s instructions.

### Immunohistochemistry

Mouse brains were fixed with 4% formaldehyde overnight at 4°C, rinsed with PBS, successively dehydrated with 10%, 20%, 30% sucrose solutions, embedded in optimized cryo-sectioning medium, sectioned, and placed onto poly-l-lysine coated slides. Sections were then incubated in 3% H_2_O_2_ for 15 min to inhibit endogenous peroxidases, washed 3 times with PBS, and incubated in blocking buffer (0.3% Triton-X-100, 5% serum in PBS) for 1 h. Sections were incubated with anti-tyrosine hydroxylase (1:200, #P40101) or GFAP (1:1,000, #16825-1-AP, Proteintech) antibodies overnight at 4°C. Subsequently, sections were incubated with HRP-labeled secondary antibody and signaled with 3, 3′-diaminobenzidine (DAB) as a substrate. After mounting on glass slides, sections were successively dehydrated in ethanol (50%, 75%, 85%, 95% and 100%), washed with xylene and coated with resinene. Microscopic imaging of TH or GFAP-positive cells was performed and the number of cells per image was manually counted by marking points in the software Image-Pro Plus 6.0 (Media Cybernetics, Inc.) (Battaglia et al., 2006). The number of cells was divided by the area of the image to obtain the number of cells per square millimeters.

### Western Blotting

Proteins were extracted from the substantia nigra and corpus striatum using RIPA lysis buffer (#P0013B, Beyotime Biotechnology). After measuring protein concentrations using the BCA assay (#P0011, Beyotime Biotechnology), equal amounts of protein from each sample were separated by SDS-PAGE gels and transferred to a nitrocellulose membranes. After blocking with 5% skim milk for 1 h at room temperature, the membranes were incubated separately with primary antibodies. Primary antibodies specific for Akt (#AF0045), phospho-Akt (Ser473) (#AF1546), Erk1/2 (#AF1051), phosphor-Erk1 (Thr202/Tyr204)/Erk2 (Thr185/Tyr187) (#AF 1891), Bcl-2 (#AF0060), *β*-actin (#AF0003), phosphor-TrkB (Tyr817) (#AF 1963) and RIP3 (#AF7893) were provided by Beyotime Biotechnology. Anti BDNF (#47808), TrkB (#4603), cleaved-Caspase-3 (#9664), TBK1 (#3504), phosphor-TBK1 (#5483), RIP1 (#3493), phospho-RIP1 (Ser166) (#31122) antibodies were provided by Cell Signaling Technology, Inc. Anti LC-3 (#bs-8878R), Beclin1 (#bs-1353R) and tyrosine hydroxylase (TH) (#bs-0016R) antibodies were provided by Beijing Biosynthesis Biotechnology. Anti GFAP (#16825-1-AP), MAP2 (#17490-1-AP) and MLKL (#21066-1-AP) were purchased from Proteintech Group, Inc. Anti FGF2 antibody (#sc-136255) was purchased from Santa Cruz Biotechnology and anti phospho-MLKL (#MABC1158) was purchased from Sigma-Aldrich. WB images were captured by an Odyssey CLx infrared fluorescence imaging system (LI-COR Biosciences). The optical density of the protein band was quantified by ImageJ software and normalized to *β*-actin.

### Statistical Analysis

Data were showed as mean ± s.e.m and plotted in GraphPad Prism 8.0 software. One-way ANOVA (analysis of variance) was used to determine statistical significance between groups, followed by Dunnett’s multiple comparisons test. For non-parametric tests, the Mann-Whitney test was applied. *, **, and *** denote *p* < 0.05, *p* < 0.01, or *p* < 0.001, respectively.

## Results

### CE Attenuates MPTP-Induced Neurotoxicity and Improves Dyskinesia in Mice

**FIGURE 1:**
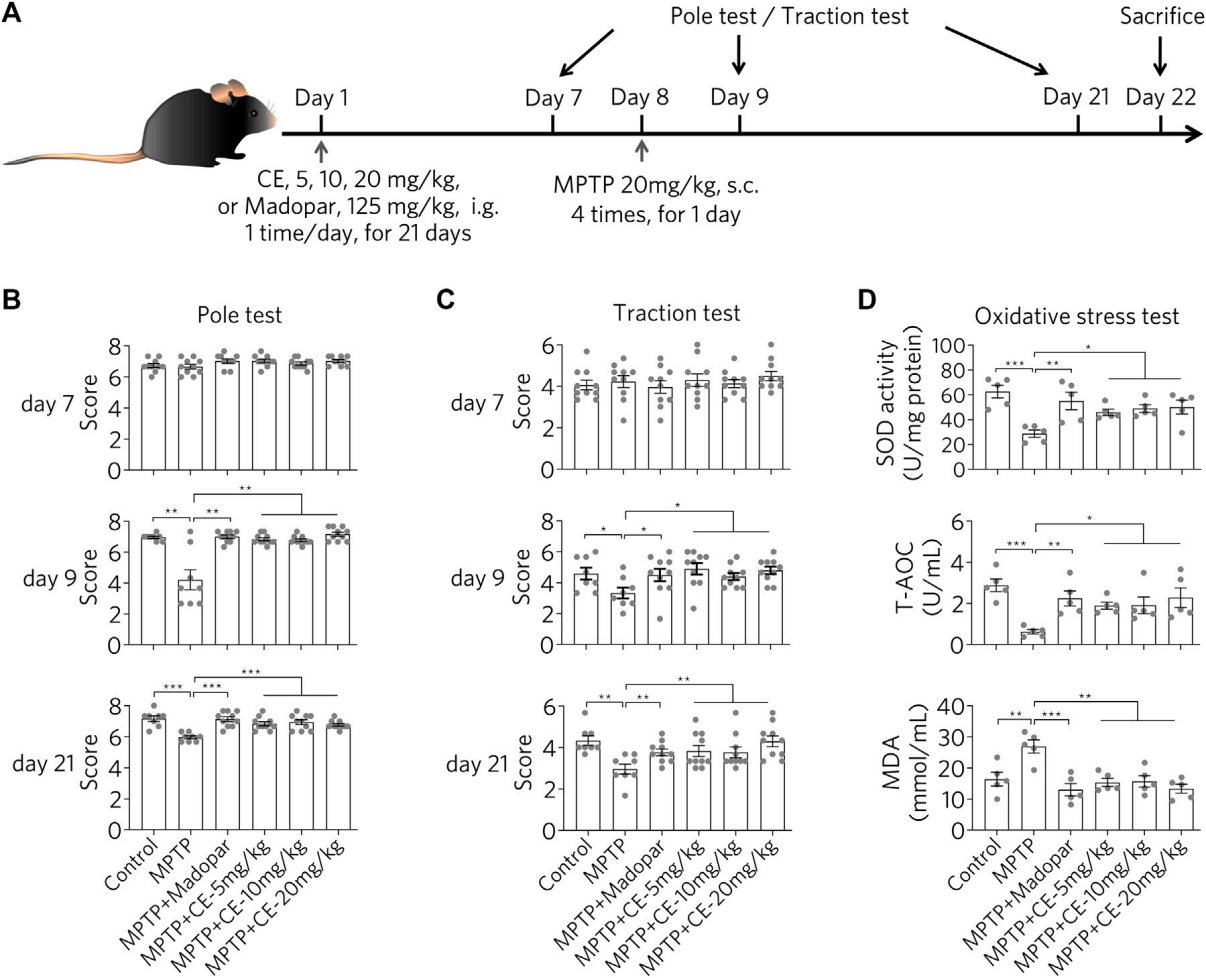
CE attenuates MPTP-induced neurotoxicity in mice. **(A)** Experimental flow of the MPTP-induced PD mouse model. **(B,C)** Behavioral analyses were performed at days 7, 9 and 21, including the pole test and traction test. Non-parametric analyses were determined by Mann-Whitney test, *n* = 10. **(D)** CE attenuates oxidative stress and promotes MDA clearance in substantia nigra. One-way ANOVA with post hoc Dunnett’s test, *n* = 5.

After injection of parkinsonian toxin MPTP (20 mg/kg, 4 times in 1 day), C57BL/6 mice exhibited motor deficits as determined by the pole test and traction test (Figure 1A), as MPTP-intoxicated mice received significantly fewer scores in behavioral analysis (Figures 1B,C, MPTP). In contrast, mice in the control and CE- or Madopar-treated groups showed resistance to MPTP-induced toxicity, as they scored similarly in the test (Figures 1B,C, MPTP + CE or MPTP + Madopar). Thus, CE was able to protect mice from the toxicity of MPTP. In addition, MPTP induced oxidative stress in the brain as total antioxidant capacity (T-AOC) and SOD decreased significantly, corresponding to the increase in the peroxidation product MDA (Figure 1D, MPTP). However, the decrease in T-AOC and SOD was alleviated by CE or Madopar treatment and facilitated the clearance of MDA (Figure 1D), so that the redox state returned to normal compared to the control. Taken together, CE attenuates MPTP-induced toxicity as well as oxidative effects and maintains its physiological function.

### CE Prevents the Loss of Dopaminergic Neurons in MPTP-Induced PD Mouse Model

**FIGURE 2:**
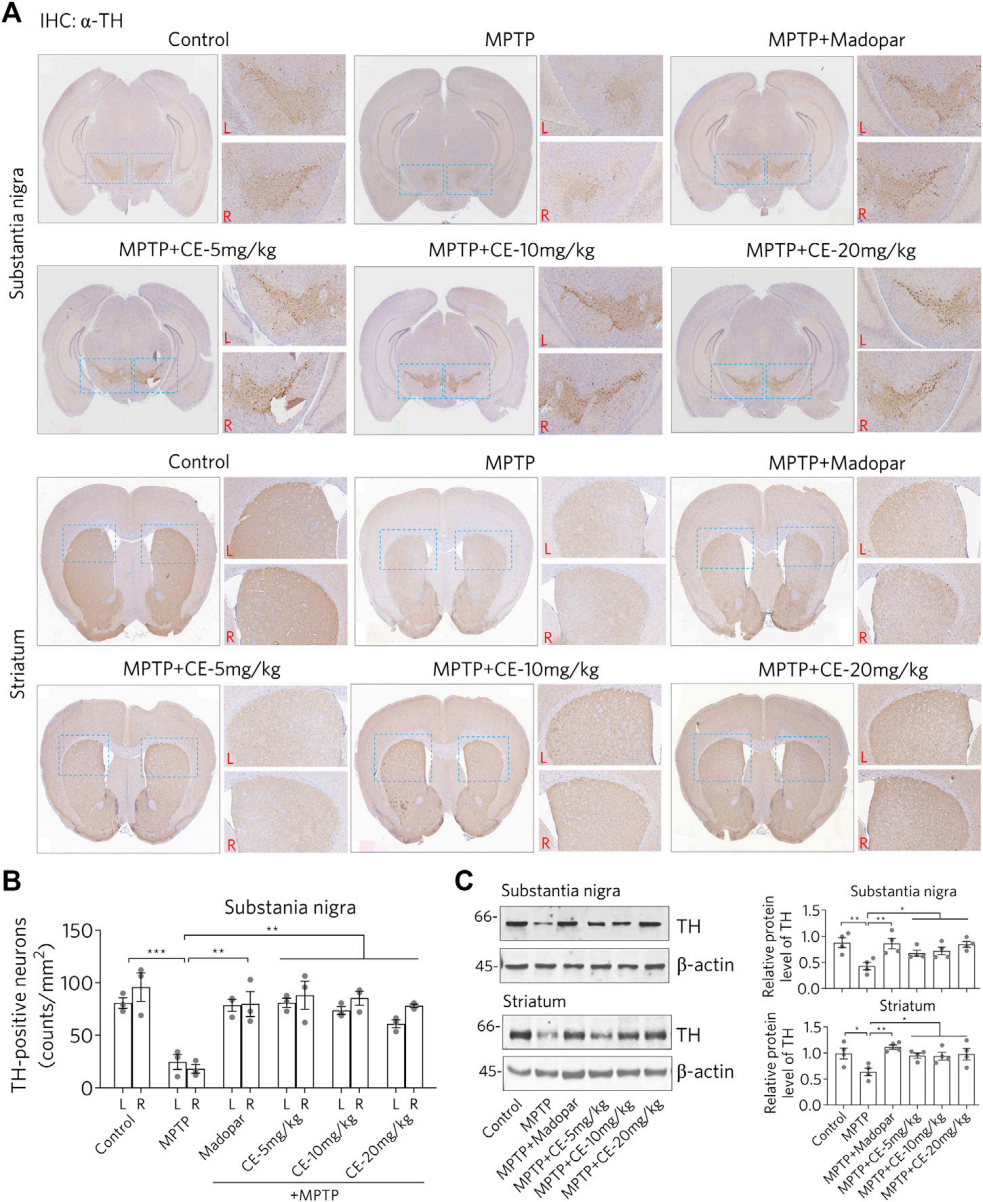
CE attenuates MPTP-induced loss of dopaminergic neurons in substantia nigra and striatum. **(A)** IHC analysis of nigrostriatal TH-positive cells. Scale bar, 1 mm. L, left; R, right. Statistical differences between groups were calculated by one-way ANOVA and post hoc Dunnett’s test, *n* = 3. **(B)** Counts of TH-positive cells. **(C)** Western blotting of TH protein. Relative protein levels were measured based on the density of blotted bands and normalized to *β*-actin. One-way ANOVA and post hoc Dunnett’s test were performed to verify the significance of differences between groups, *n* = 4.

Loss of dopaminergic neurons in the substantia nigra and striatum is the most characteristic feature of PD. IHC staining was performed using an antibody specific for TH to identify dopaminergic neurons in nigrostriatal sections (Figure 2). In MPTP-induced PD mice, the signal of dopaminergic neurons was remarkably reduced (Figure 2A, MPTP), while dopaminergic neurons were normally aligned in a dovetail shape, which was observed in both control and CE or Madopar treatment groups (Figure 2A, MPTP + CE or MPTP + Madopar). Likewise, the counts of dopaminergic neuron numbers on IHC sections confirmed the MPTP-induced reduction and CE- or Madopar-mediated recovery (Figure 2B). Furthermore, protein levels of TH were determined by Western blotting. As shown in Figure 2C, TH was significantly reduced by MPTP treatment, but was rescued by CE- or Madopar-treatments. Therefore, CE was able to inhibit the loss of dopaminergic neurons induced by MPTP. In other words, the anti-PD effect of CE was similar to that of Madopar, a drug used clinically.

### CE Attenuates MPTP-Induced Astrocyte Activation and Inflammation to Protect Neurons

**FIGURE 3:**
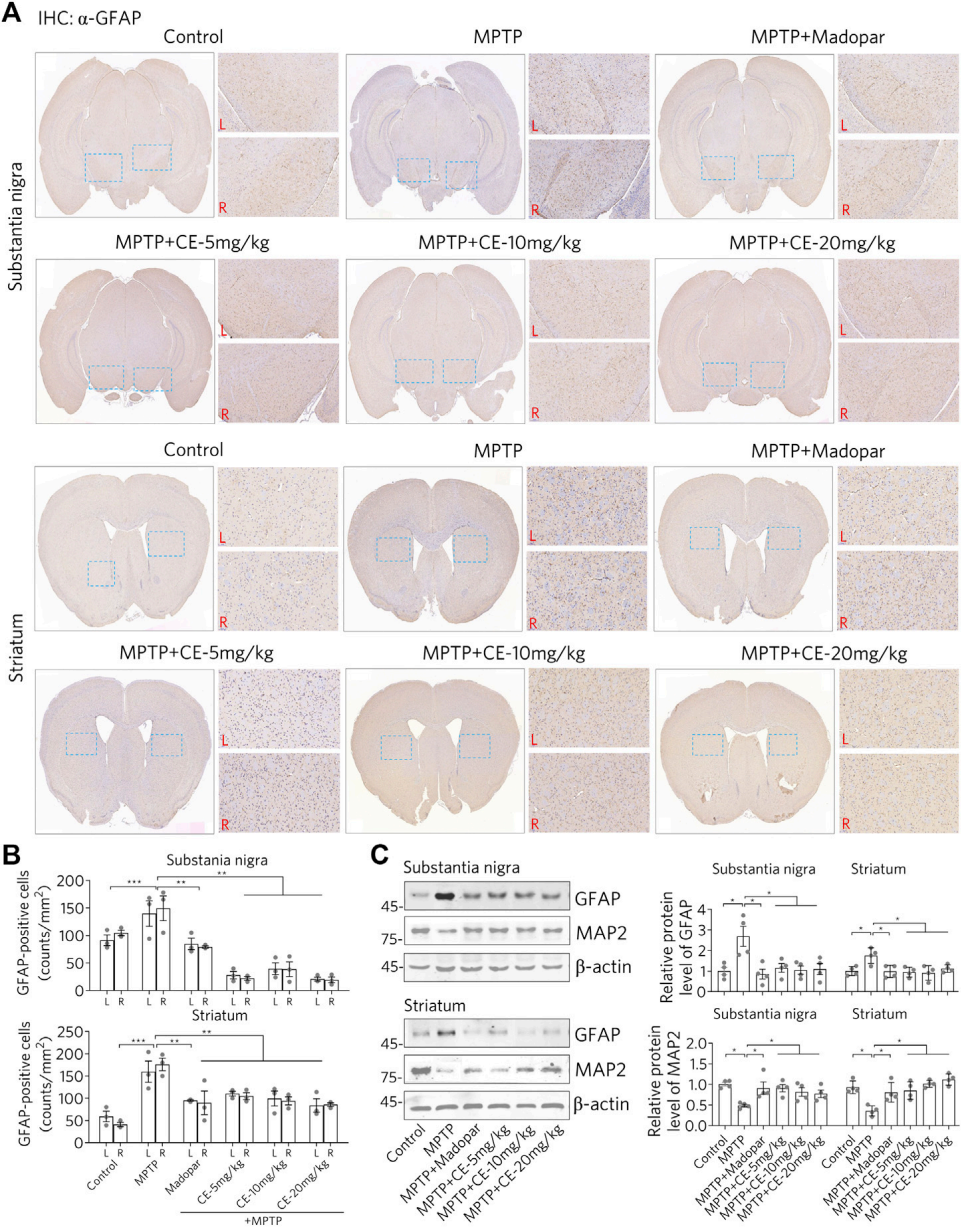
CE attenuates astrocyte activation and protects neurons from MPTP-induced toxicity. **(A)** IHC staining of GFAP-positive cells in substantia nigra and striatum. **(B)** Counts of TH-positive cells, *n* = 3. **(C)** Western blotting of GFAP and MAP2 proteins. Relative protein levels were examined according to the density of blotted bands and normalized to *β*-actin, *n* = 4. One-way ANOVA, post hoc Dunnett’s test was performed to verify the significance of differences between groups.

As shown in Figure 2, MPTP leads to the loss of dopaminergic neurons, probably through apoptosis. Glial cells are activated when the damaged cells are processed. It was after injection of MPTP that we observed activation of astrocytes in the substantia nigra and striatum, as evidenced by IHC staining using an antibody specific for GFAP, a marker protein for astrocytes (Figure 3A, MPTP). However, the activation of astrocytes was attenuated under CE or Madopar treatment (Figure 3A, MPTP + CE or MPTP + Madopar). Changes in the number of GFAP-positive cells in the substantia nigra and striatum also confirmed the results of IHC staining, as CE or Madopar treatment attenuated the MPTP-induced increase in GFAP-positive cells (Figure 3B). In addition, the changes in GFAP-positive cells were replicated by Western blotting to detect GFAP protein (Figure 3C). In detail, the levels of GFAP protein in the substantia nigra and striatum were significantly increased after MTTP intoxication (Figure 3C, MPTP). The increase in GFAP protein levels was mitigated after treatment with CE or Madopar. In addition, the protein marker MAP2 in neurons showed antagonistic changes compared to GFAP, but consistent with changes in dopaminergic neurons. That is, the MPTP-induced decrease in MAP2 protein corresponded to the loss of dopaminergic neurons, whereas the recovery of MAP2 corresponded to the maintenance of dopaminergic neurons (Figure 3C, MAP2). Thus, CE prevents MPTP-induced nigrostriatal atrophy and maintains cellular homeostasis in the brain.

**FIGURE 4:**
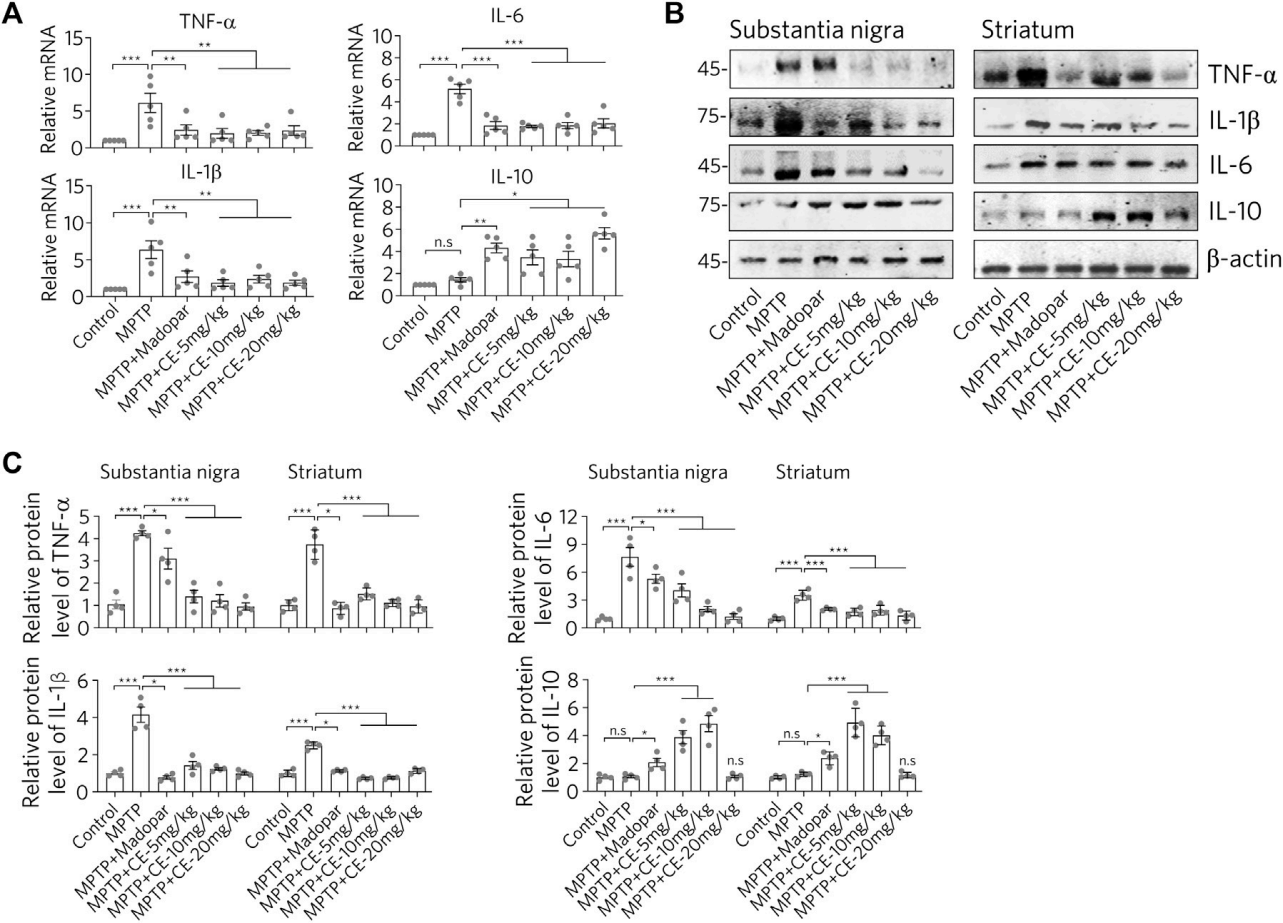
CE restores elevated levels of pro-inflammatory factors. **(A)** RT-PCR determination of mRNA levels of pro-inflammatory factors in substantia nigra, *n* = 5. **(B)** Western blotting analysis of pro-inflammatory factors and anti-inflammatory factor IL-10. **(C)** Relative protein levels normalized to *β*-actin, *n* = 4. In A and C, one-way ANOVA followed by Dunnett’s test were performed.

Consistent with the changes in astrocytes status, there was a corresponding response to typical pro-inflammatory factors (Figure 4). After MPTP-treatment, we observed increased mRNA and protein levels of TNF-α, IL-1β and IL-6 (Figure 4, MPTP). Interestingly, IL-10, an anti-inflammatory factor, was down regulated (Figure 4, MPTP column for IL-10). These results suggest that MPTP intoxication in the CNS triggers a pro-inflammatory response. However, under CE or Madopar treatment, increases in pro-inflammatory factors including TNF-α, IL-1β, and IL-6 were suppressed, while IL-10 was recovered (Figure 4, MPTP + CE or MPTP + Madopar). Thus, we conclude that CE attenuates MPTP-induced inflammation in the substantia nigra and striatum.

CE maintains the activity of the BDNF-TrkB and FGF2-Akt signaling pathways and inhibits apoptosis and autophagy, thereby attenuating MPTP-induced neurotoxicity.

**FIGURE 5:**
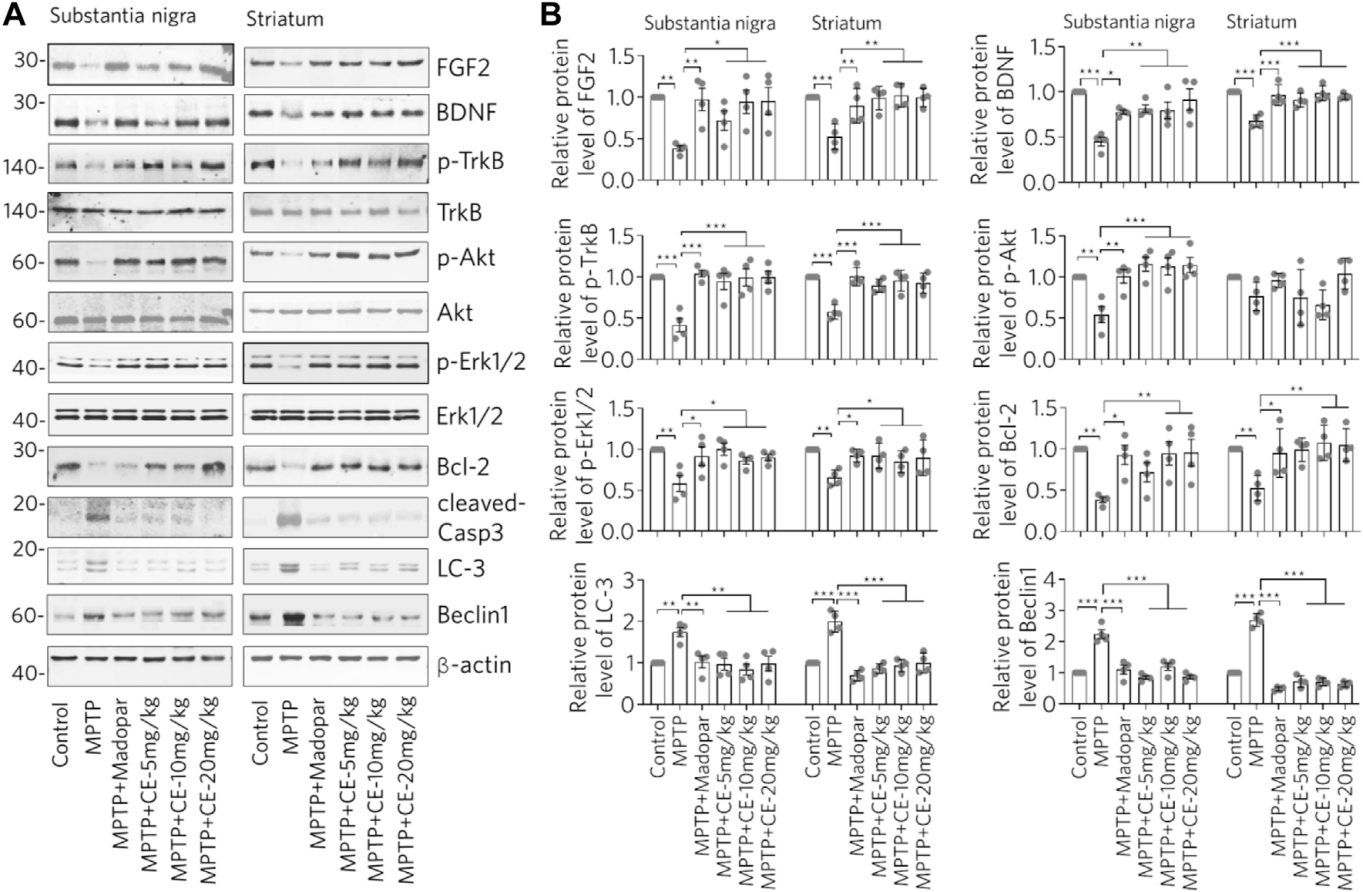
CE maintains the BDNF-TrkB and FGF2-Akt signaling pathways and inhibits apoptosis and autophagy to attenuate MPTP-induced toxicity. **(A)** Western blotting analysis of BDNF, FGF2 and their mediated signaling pathways, as well as marker proteins for apoptosis and autophagy. **(B)** Relative protein levels were calculated from the density of blotted bands and normalized to *β*-actin. One-way ANOVA with post hoc Dunnett’s test, *n* = 4.

We have previously shown that the FGF2-PI3K/Akt and BDNF-TrkB signaling pathways are required for neural survival and activity (Zhong et al., 2019). Therefore, we wanted to ask whether BDNF, FGF2 and their downstream signaling regulators are involved in CE-mediated anti-PD activity. In MPTP-intoxicated mice, we found significantly decreased protein levels of BDNF and FGF2 in the substantia nigra and striatum (Figure 5A, MPTP). Accordingly, the TrkB/Akt-Bcl-2 signaling pathway regulated by BDNF and FGF2 was inhibited. In detail, the active forms of TrkB and Akt indicating by their phosphorylation were reduced. Similar change was observed for Erk1/2, another kinase that regulates cell proliferation and apoptosis. In addition, the anti-apoptosis protein Bcl-2 was down regulated (Figure 5A, Bcl-2). In contrast, the active form of the apoptotic enzyme Caspase-3 (cleaved Casp-3) was increased (Figure 5A, cleaved-Casp-3). However, the BDNF-TrkB/Akt-Bcl-2 and Erk1/2 signaling pathways were restored under CE or Madopar treatment, as the aforementioned proteins were restored. In particular, the antagonistic change of Bcl-2/cleaved-Casp-3 clearly implied the inhibition of apoptosis (Figure 5A). The above changes were also confirmed by the relative protein levels calculated from the optical densities of the blotted bands (Figure 5B). In addition, we examined LC-3 and Beclin1, two proteins that are indicators of autophagy. We found that both LC-3 and Beclin1 increased after MPTP-intoxication (Figure 5, LC-3 and Beclin1). In other words, MPTP-induced atrophy of nigrostriatal neurons and induced the activity of autophagy, which may be required for the removal of damaged cells and substances. In contrast, CE or Madopar attenuated the increase in LC-3 and Beclin1, suggesting a restoration of cellular homeostasis. From these results, we found that CE was able to restore the levels of BDNF and FGF2, reactivate their downstream signaling axes, and inhibit apoptosis and autophagy. Also, BDNF-TrkB and FGF2-Akt signaling pathways are essential for CE-mediated anti-PD function.

### CE Alleviates the RIP1-Driven Inflammatory Pathway in the MPTP-Induced PD Mice Model

**FIGURE 6:**
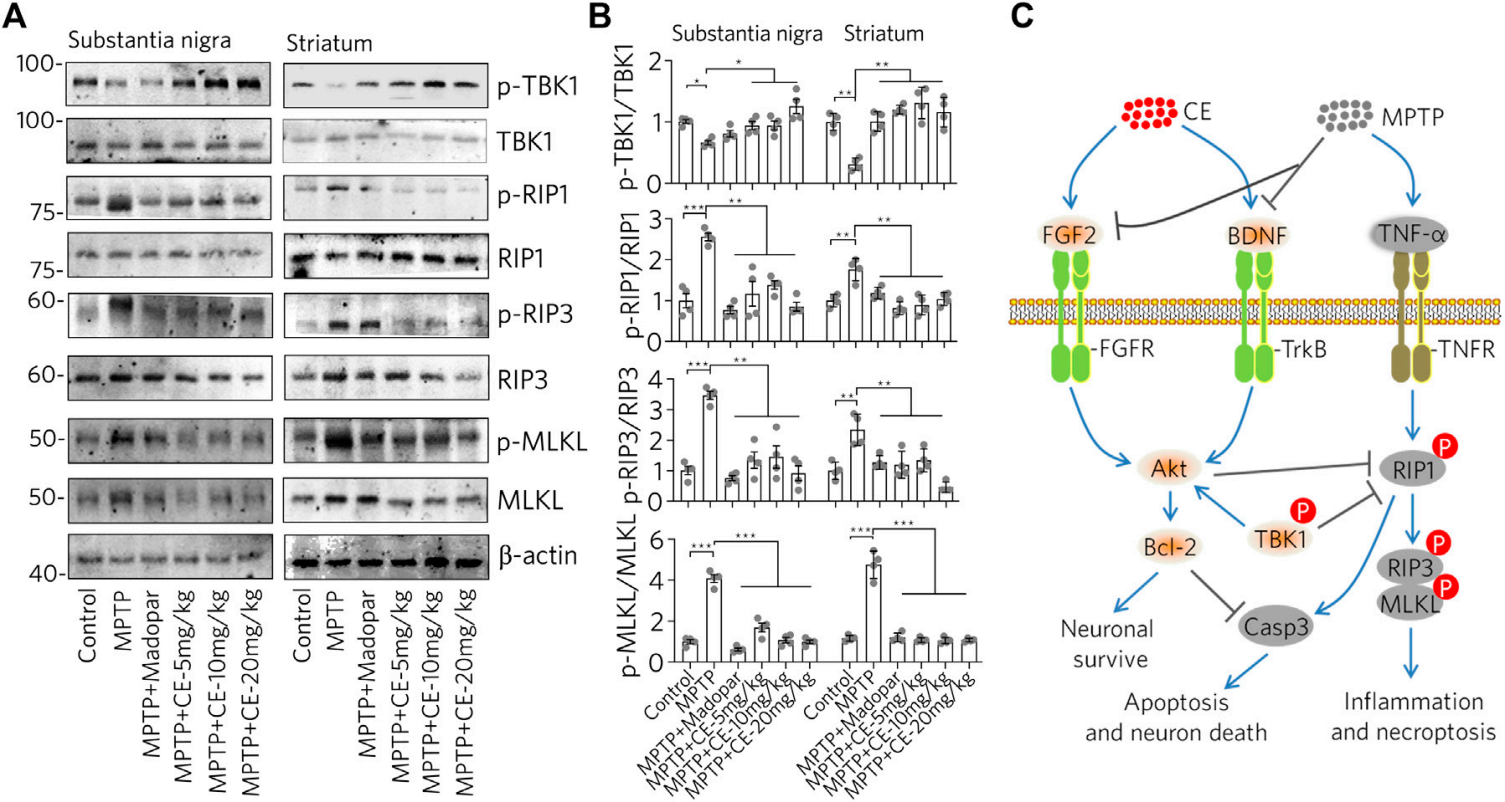
CE inhibits the RIP1-driven inflammatory pathway triggered by MPTP-induced neurotoxicity. **(A)** Western blot of RIP1-driven inflammatory pathways in substantia nigra and striatum. **(B)** Statistical analysis of protein levels based on the blots in **(A)** and normalized to *β*-actin. One-way ANOVA wihtc Dunnett’s test, *n* = 4. **(C)** Schematic representation of the anti-PD effects mediated by CE in a mouse model of MPTP-induced PD. In the brain, MPTP impaired the levels of BDNF, FGF2 and its regulated signaling pathways, as well as the RIP1-driven inflammatory pathway, leading to apoptosis of dopaminergic neurons. However, CE attenuated MPTP-induced toxicity and abnormalities in the above-mentioned signaling pathways to protect neurons.

Consistent with the typical inflammation mediated by glial cell activation and release of pro-inflammatory factors (Figures 3, 4), we found that the RIP1-driven inflammatory pathway was also activated after MPTP-intoxication (Figure 6A, MPTP). In detail, RIP1, RIP3 and MLKL along with their phosphorylated forms, were increased with MPTP treatment, indicating activation. Correspondingly, TBK1, an endogenous inhibitor of RIP1 was reduced. Similar to the changes in the BDNF-TrkB/Akt signaling axis, TBK1 was recovered with the inhibition of RIP1/RIP3/MLKL by CE or Madopar treatment, and all three proteins were down regulated (Figure 6A, MPTP + CE or MPTP + Madopar). Computational and statistical analysis of protein band density confirmed the observed changes (Figure 6B). Thus, CE is able to inhibit RIP1-driven inflammatory pathway.

In summary, we explored the anti-PD effects of CE in an MPTP-induced mouse model (Figure 6C). The BDNF-TrkB and FGF2-Akt signaling axes are involved in inhibiting apoptosis and maintaining neuronal survival. After MPTP intoxication, BDNF and FGF2 were inhibited, while the TrkB/Akt signaling pathway is inactive, accompanied by apoptosis and RIP1-driven inflammation. In contrast, under CE treatment, the levels of BDNF and FGF2 and their regulated signaling pathways were restored, leading to inhibition of apoptosis and attenuation of inflammation.

## Discussion

Genetically or environmentally induced loss of nigrostriatal neurons in the brain is a major feature of PD pathology and has been confirmed in studies of MPTP-intoxicated mice (Figures 2, 3). Maintenance of cellular homeostasis or rescue of neuronal loss should contribute to the treatment of PD. Currently, the treatment of PD is focused on controlling symptoms, delaying the progression of the disease and improving the quality of life of patients. Levodopa (L-3,4-dihydroxyphenylalanine, called levodopa), dopamine agonists, monoamine oxidase B inhibitors, and anticholinergic agents are commonly used to treat PD. Otherwise, cell transplantation is another approach to treat PD, where cells are injected into the substantia nigra in the hope of replacing the dopamine-producing neurons that have been lost (Chao et al., 2016). However, obtaining suitable transplanted cells and avoiding side effects are major obstacles that need further research. Moreover, natural products, especially medicinal plants used in ethnomedicine, have been accumulated and practiced for thousands of years, providing a rich resource for drug development, especially for drugs with good neuromodulatory activity that have been clinically and ethnically validated. In fact, we have previously demonstrated the antioxidant, anti-inflammatory, anti-aging and anti-AD effects of CE in cell-based *in vitro* and *in vivo* mouse models (Pan et al., 2017; Zhong et al., 2019; Li et al., 2021; Li et al., 2022). Thus, it is very useful to investigate the anti-PD effects of CE in this work. With reference to the establishment of experimental PD models, MPTP is a widely used and reused agent that induces the loss of dopaminergic neurons through its toxic metabolite MPP+ in the brain (Dauer and Przedborski, 2003). Therefore, we used the MPTP-induced PD model in mice to assess the anti-PD activity of CE. To investigate the protective effects of CE on nigrostriatal neurons, we focused on signaling pathways related to cell survival and apoptosis, such as PI3K/Akt and Bcl-2/Caspase-3. Prevailing evidence supports the role of BDNF, FGF2 and its regulated TrkB/Akt, MAPK and PLC signaling axes in regulating neuronal survival and maintaining cellular homeostasis (Yoshii and Constantine-Paton, 2007; Franke, 2008). Consistently, insufficient expression of BDNF and impairment of the BDNF-TrkB signaling axis are frequently observed in AD, PD and even chronic stress-induced depression (Martinowich et al., 2007; Lu et al., 2013; Zhong et al., 2019). At the mechanistic level, the positive feedback loop of BDNF-TrkB signaling depends on CREB and C/EBPβ mediated-transcription, which is crucial for neuronal survival and synaptic plasticity (Bambah-Mukku et al., 2014; Kowianski et al., 2018). Indeed, we found in this work that MPTP-intoxication induced a significant reduction in BDNF, FGF2 and their related signaling axis, corresponding to the activation of apoptosis-related enzyme such as cleaved-Casp-3 and the reduction of anti-apoptotic protein Bcl-2 (Figure 5). In light of this, improving the pathological state of BDNF and the signaling pathways it regulates is a promise strategy for the pharmacological treatment of CNS diseases.

In addition, the inflammatory pathway is also in focus because it is a common phenomenon and pathology of the CNS. Glial cells including microglia and astrocytes are the most abundant cells in the CNS that respond to insults from intracellular or exogenous sources and remove damaged cells, plaques and toxic substances, thereby maintaining cellular homeostasis (Salter and Stevens, 2017). On the other hand, widespread activation of glial cells adversely affects neurons by releasing pro-inflammatory factors and free radicals, and even evokes inflammatory storms that lead to neuronal damage and cell death. Moreover, the RIP1-driven inflammatory pathway is the master signal that regulates the decision between promoting survival and cell death in response to a large number of stimuli. RIP1-mediated necroptosis is accomplished through the formation of the RIP1/RIP3/MLKL complex, which is regulated by cascade phosphorylation. RIP1 activation and necroptosis have been genetically and mechanistically linked to human neurodegenerative diseases (Mifflin et al., 2020). Indeed, we found activation of astrocytes and RIP1-driven inflammation after MPTP-intoxication. Conversely, CE or Madopar could alleviate the aforementioned inflammation and normalize the cellular state (Figures 3, 4, 6). Also, predictably, RIP1 inhibitors may be chemicals of interest for PD treatment by inhibiting RIP1-mediated inflammation, and the presence of inhibitors of RIP1 in CE is an interesting question. However, identifying the direct targets or molecules directly regulated by CE that then exert subsequent anti-PD activity remains a difficulty. This is mainly due to the fact that CE has multiple metabolites in the body and the exact molecule that interacts directly with CE-derived neurons in the CNS remains unidentified. Nevertheless, CE is effective to protect dopaminergic neurons from MPTP-induced toxicity and rescued motor deficits in mice. This also suggests that BDNF, FGF2 and their associated signaling pathways may be effective targets for drug design and PD treatment. Since CE has been used since ancient times and some medical products derived from CE are already available, it is important to validate the anti-PD effects of CE in PD patients in further studies. Thus, pharmacological treatment of CE may protect nigrostriatal neurons from MPTP-mediated toxicity and may pave a new pathway for BDNF, FGF2 and TrkB/Akt signaling axis as targets for the treatment of PD.

## Data Availability

The original contributions presented in the study are included in the article/Supplementary Material, further inquiries can be directed to the corresponding authors.

## Abbreviations

BDNF, brain-derived neurotrophic factor; CE, Coeloglossum viride var. bracteatum extract; FGF2, fibroblast growth factor 2; IL-6, interleukin-6; IL-1β, interleukin-1β; MDA, malondialdehyde; MLKL, Mixed lineage kinase domain-like protein; MPTP, 1-methyl-4-phenyl-1, 2, 3, 6-tetrahydropyridine; PD, Parkinson’s disease; RIP1, receptor-interacting serine/threonine-protein kinase 1; RIP3, receptor-interacting serine/threonine-protein kinase 3; SOD, superoxide dismutase; T-AOC, total antioxidant capacity; TBK1, TANK-binding kinase 1; TH, tyrosine hydroxylase; TNF-α, tumor necrosis factor alpha.
