# Supplementary material for: Coeloglossum viride Var. Bracteatum Extract Attenuates MPTP-Induced Neurotoxicity in vivo by Restoring BDNF-TrkB and FGF2-Akt Signaling Axis and Inhibiting RIP1-Driven Inflammation
Source: Front Pharmacol. 2022 Apr 28;13:903235. doi: 10.3389/fphar.2022.903235 (PMC9096617; doi:10.3389/fphar.2022.903235)
Supplement: Supplementary file 1 [file Presentation1.PPTX]

## Slide 1
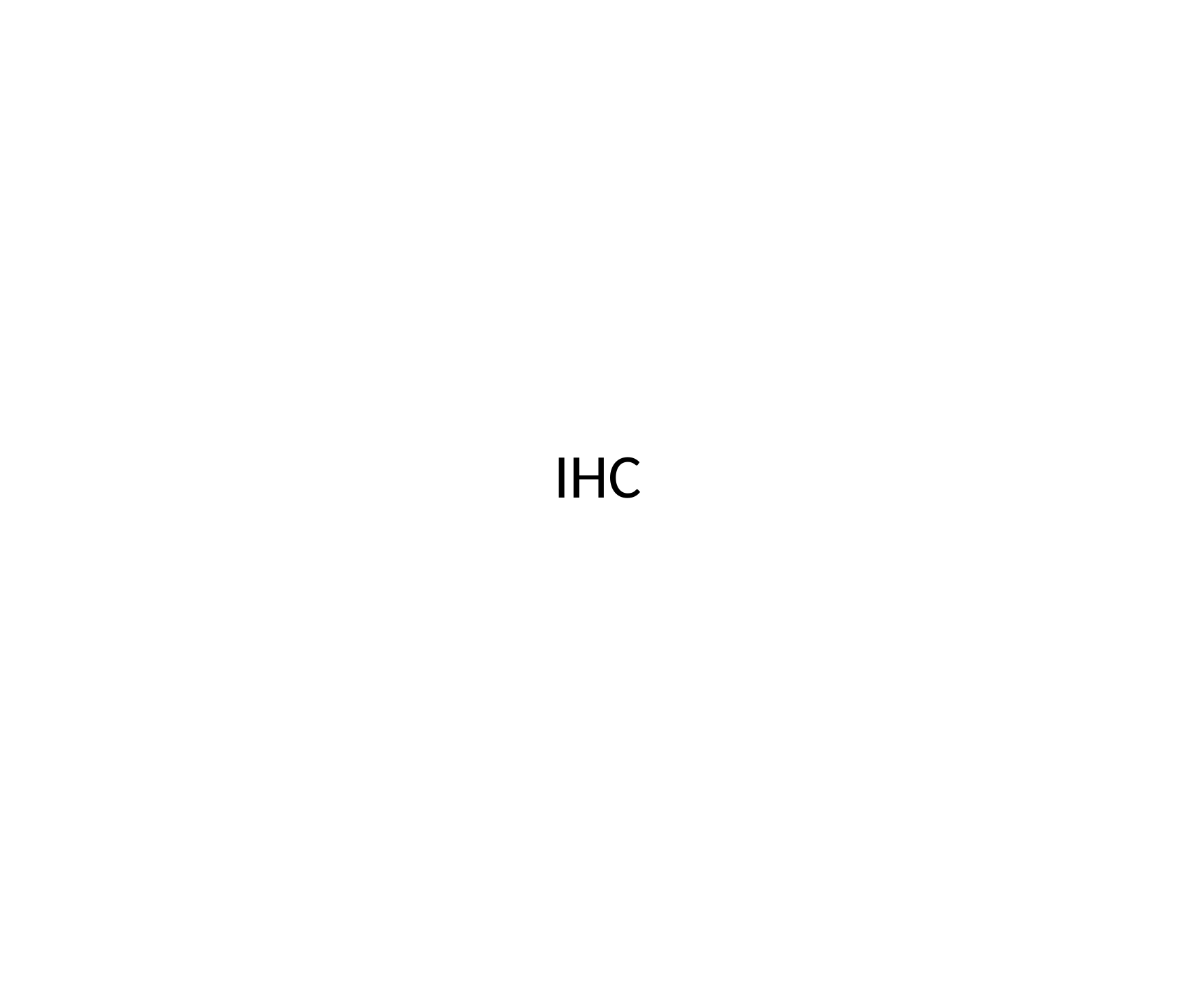

# IHC

## Slide 2
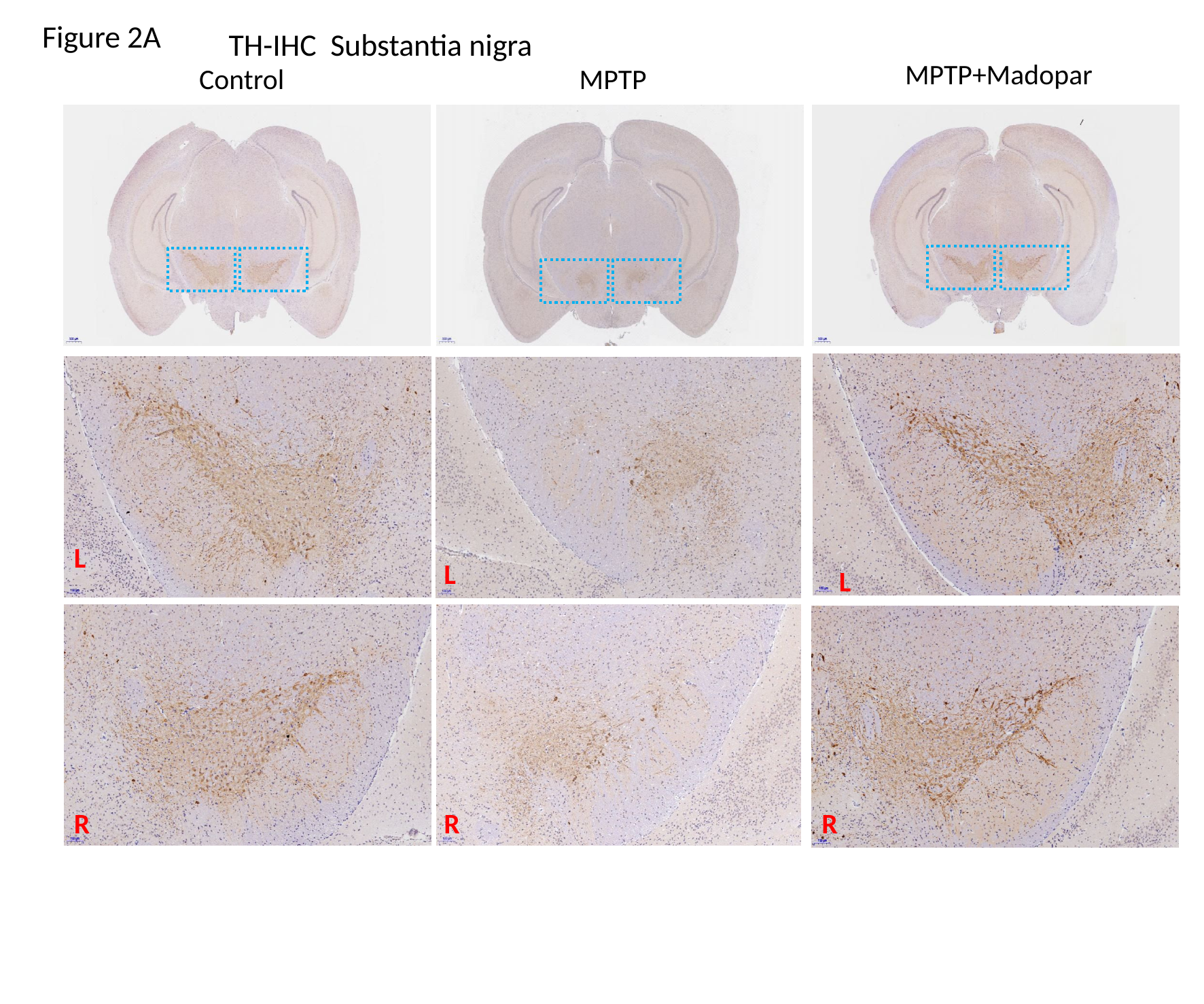

Figure 2A
TH-IHC Substantia nigra
MPTP+Madopar
Control
MPTP
L
L
L
R
R
R

## Slide 3
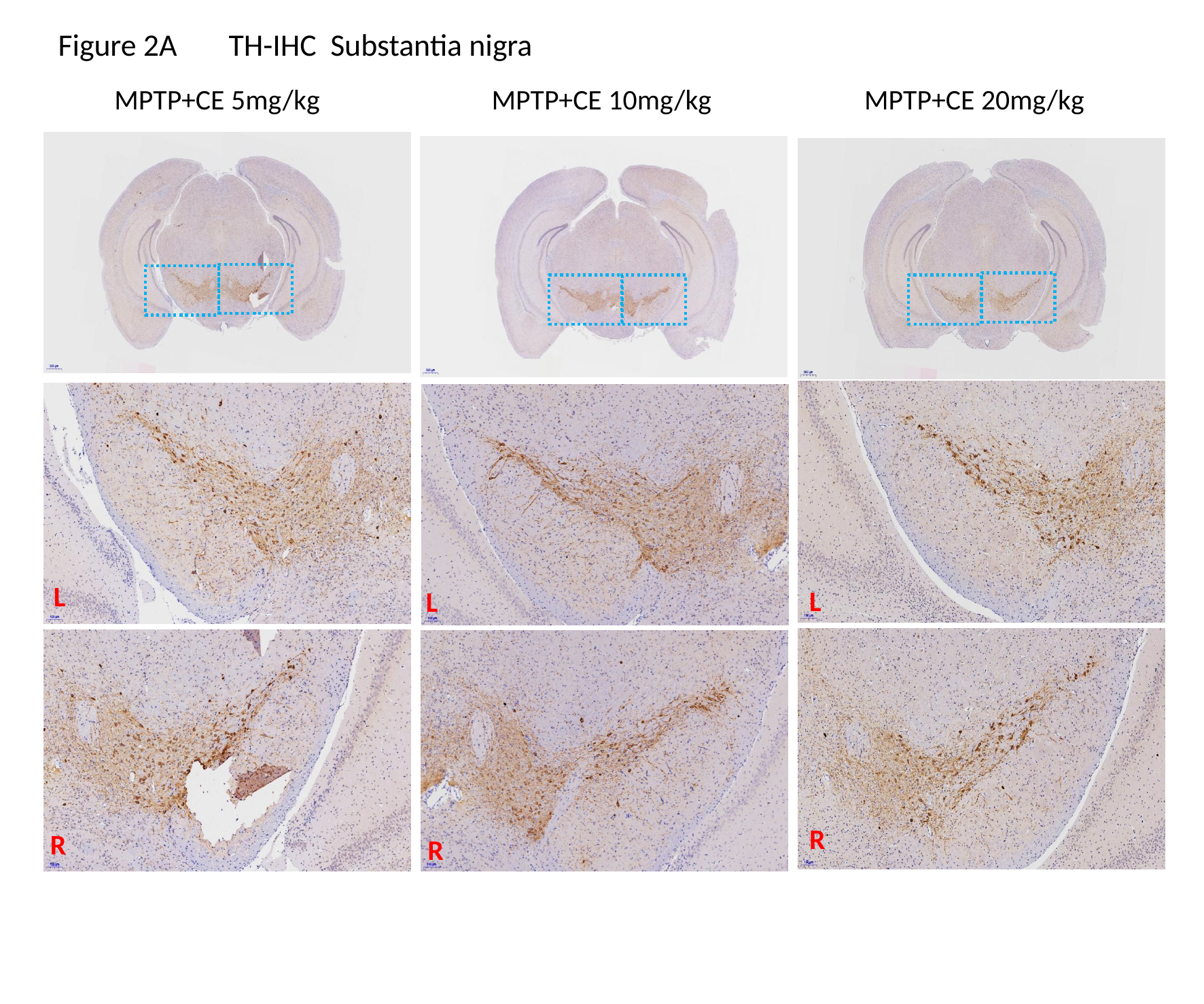

Figure 2A
TH-IHC Substantia nigra
MPTP+CE 5mg/kg
MPTP+CE 10mg/kg
MPTP+CE 20mg/kg
L
L
L
R
R
R

## Slide 4
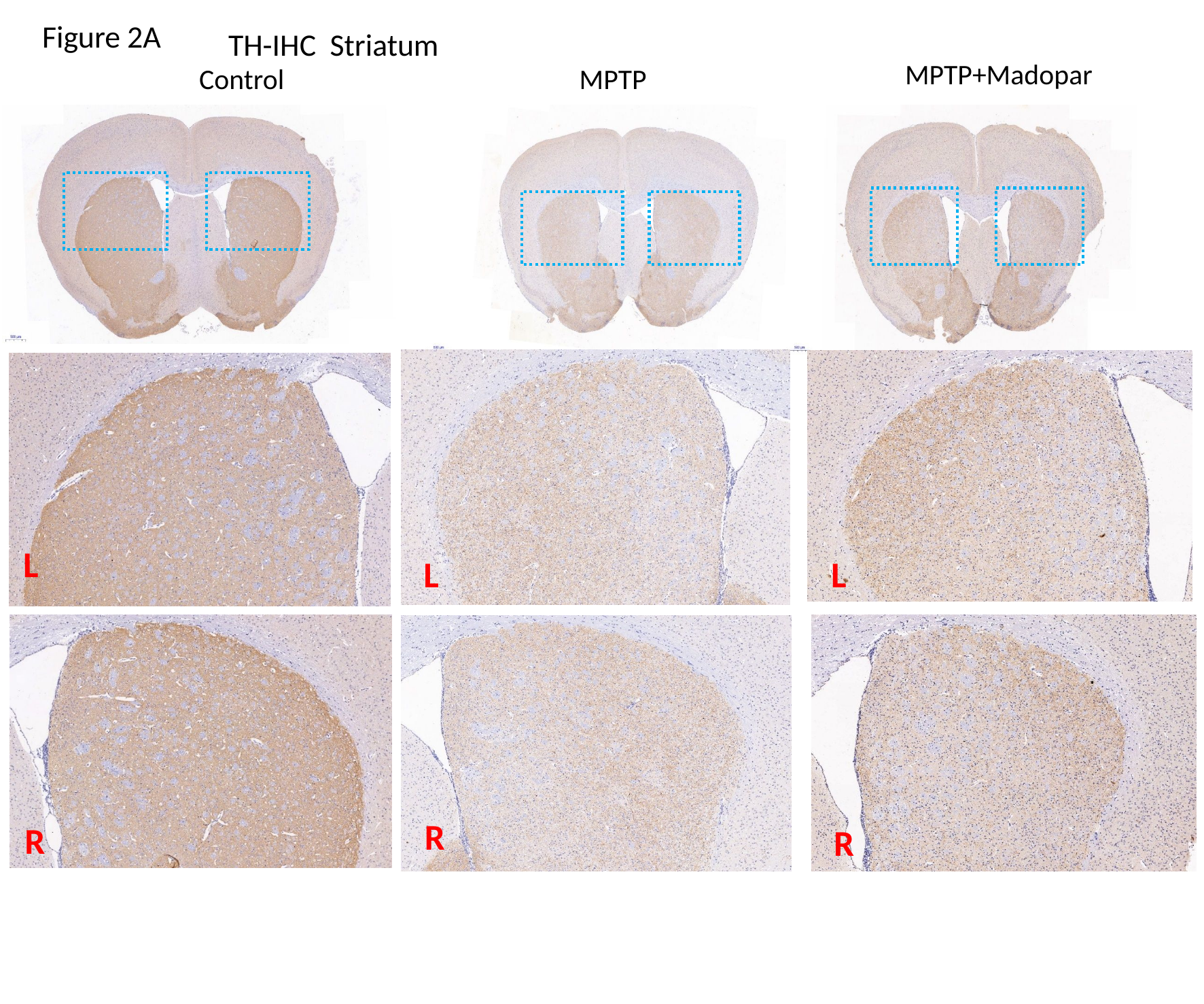

Figure 2A
TH-IHC Striatum
MPTP+Madopar
Control
MPTP
L
L
L
R
R
R

## Slide 5
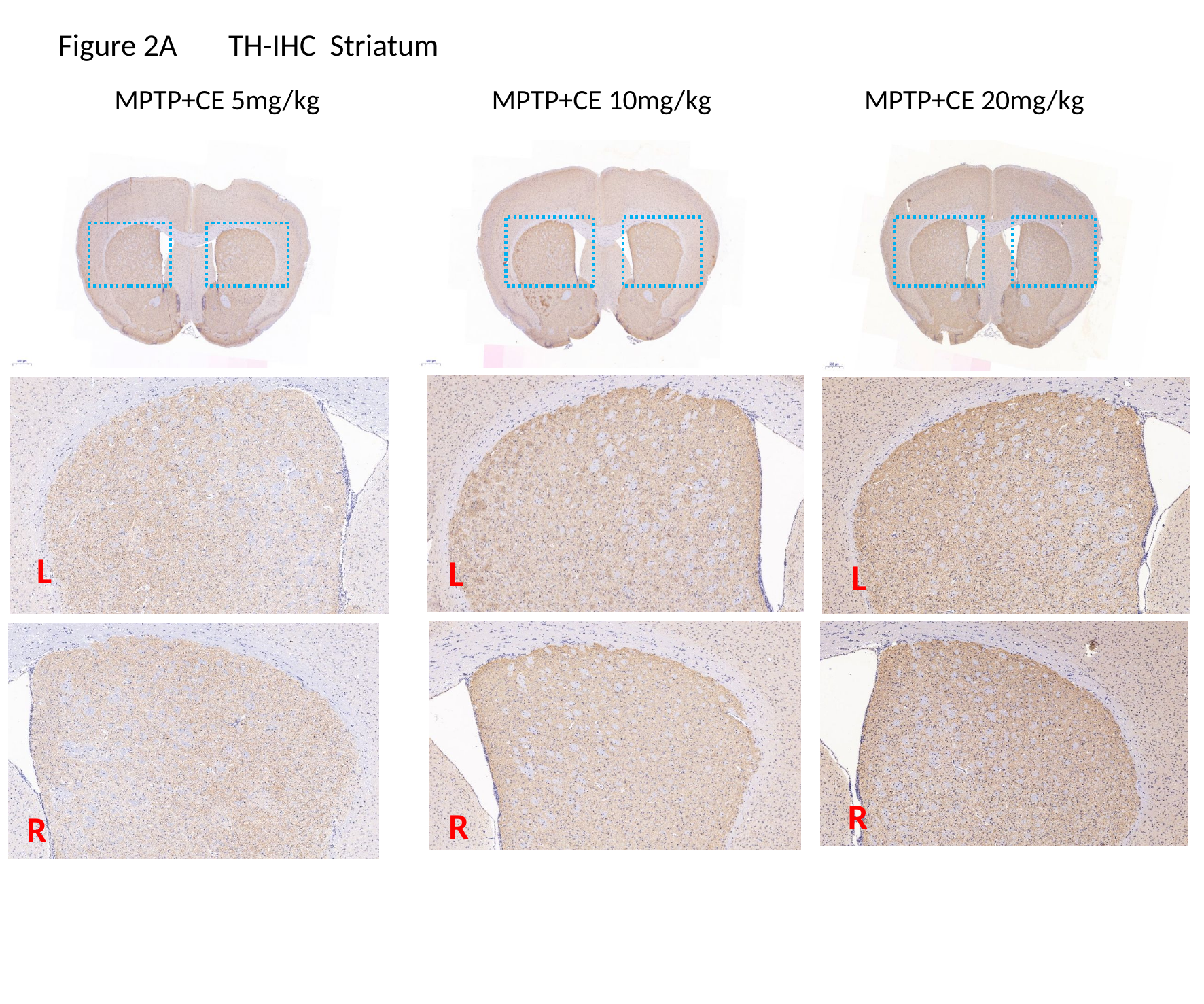

Figure 2A
TH-IHC Striatum
MPTP+CE 5mg/kg
MPTP+CE 10mg/kg
MPTP+CE 20mg/kg
L
L
L
R
R
R

## Slide 6
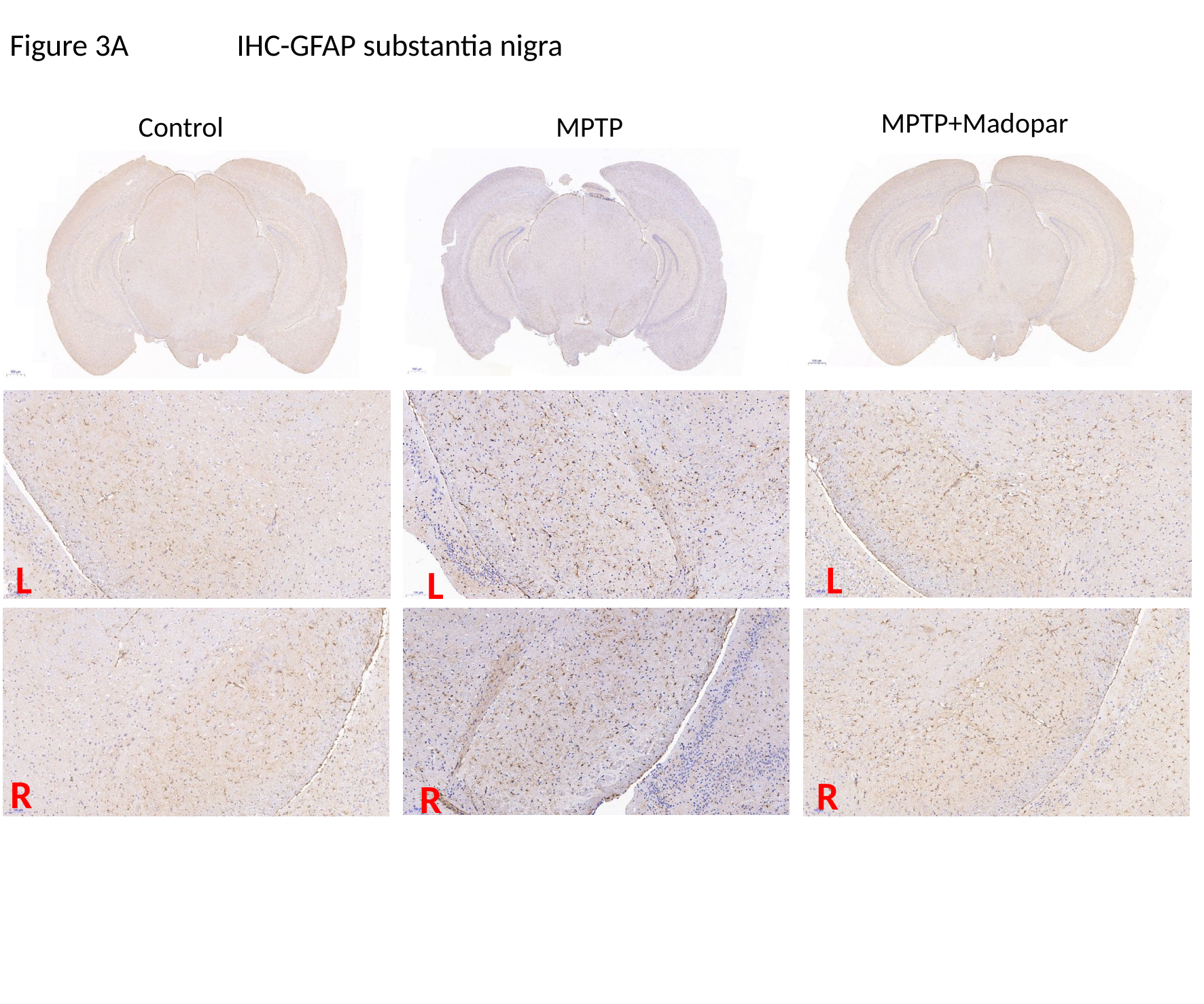

Figure 3A
IHC-GFAP substantia nigra
MPTP+Madopar
Control
MPTP
L
L
L
R
R
R

## Slide 7
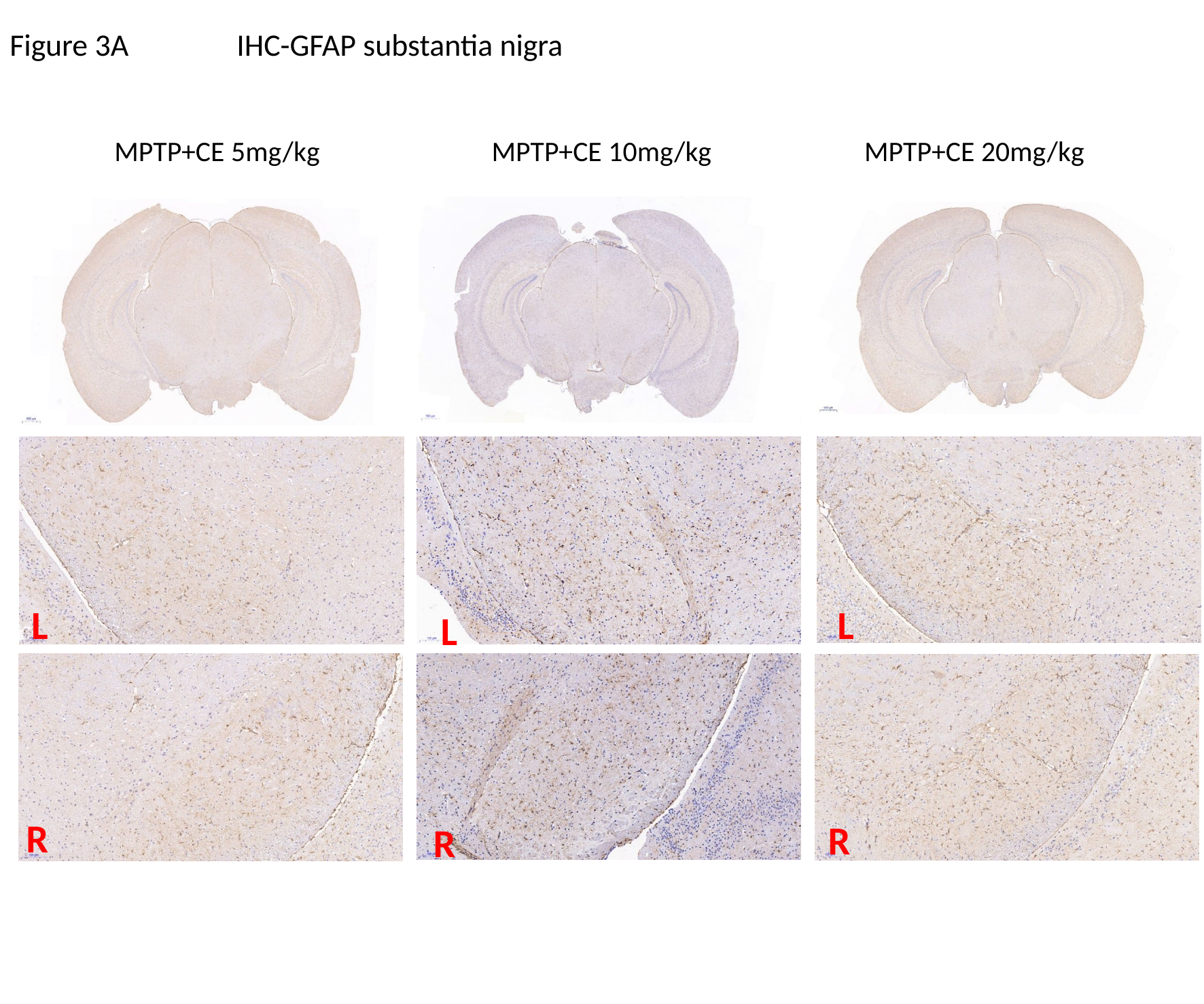

Figure 3A
IHC-GFAP substantia nigra
MPTP+CE 5mg/kg
MPTP+CE 10mg/kg
MPTP+CE 20mg/kg
L
L
L
R
R
R

## Slide 8
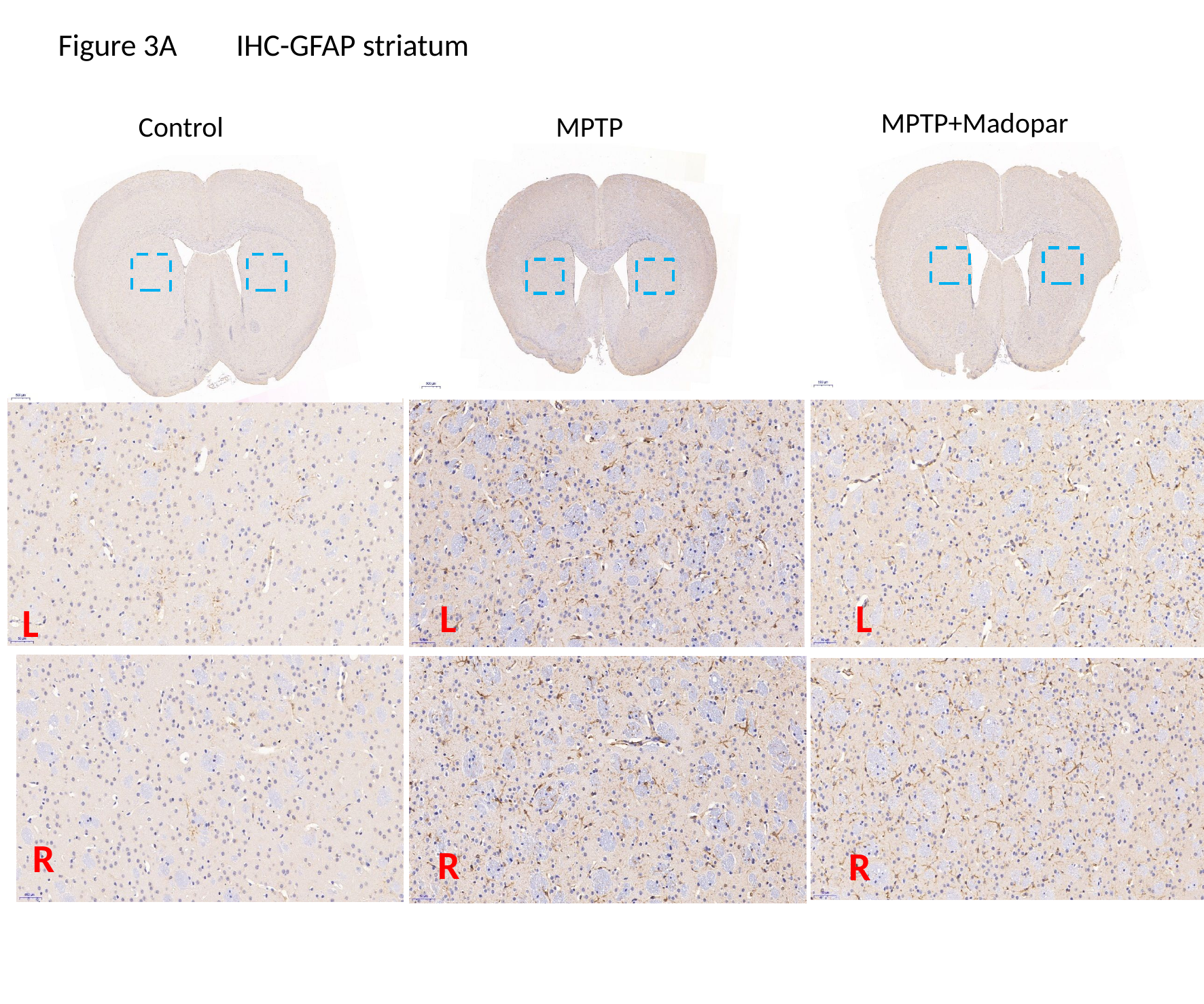

Figure 3A
IHC-GFAP striatum
MPTP+Madopar
Control
MPTP
L
L
L
R
R
R

## Slide 9
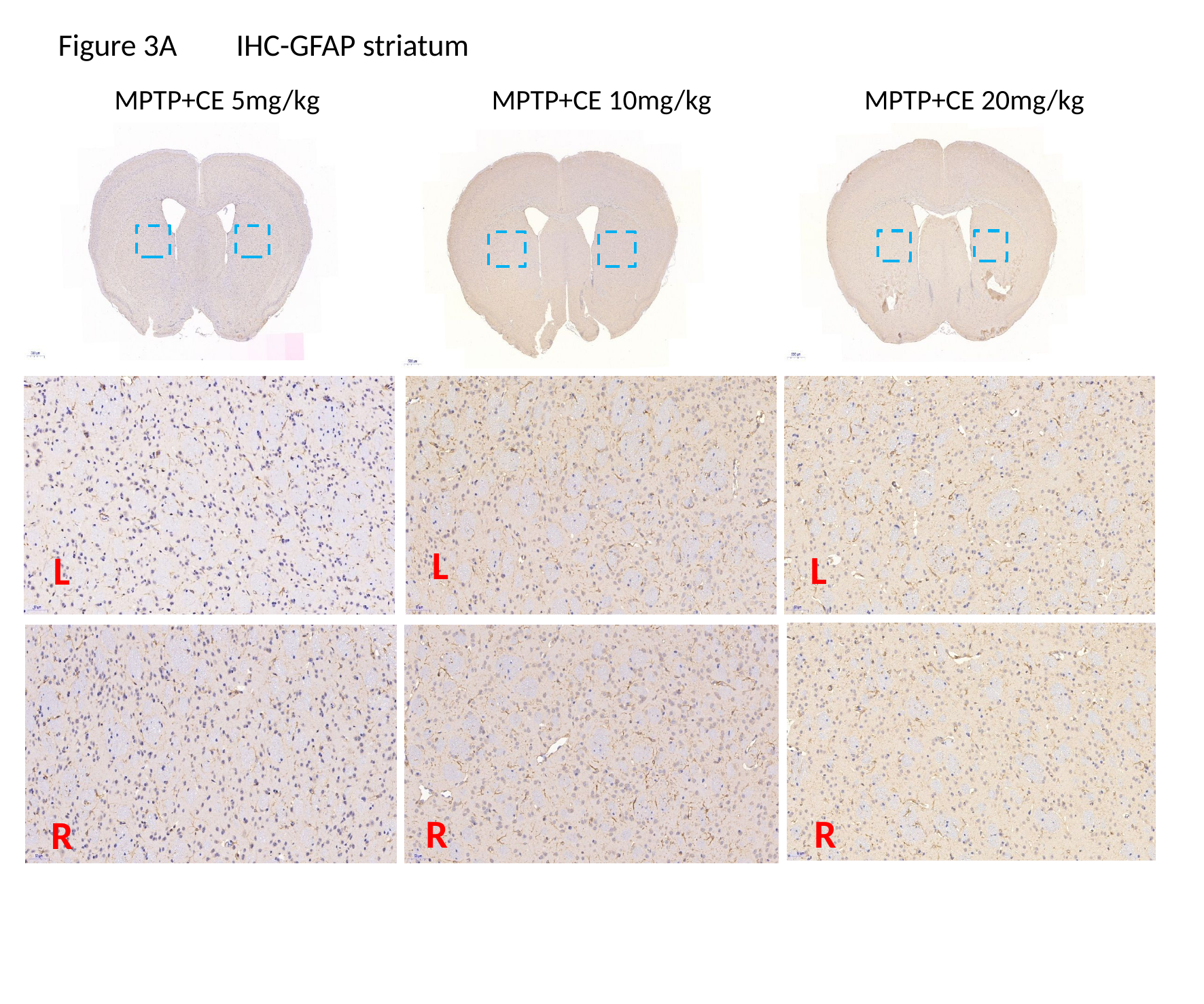

Figure 3A
IHC-GFAP striatum
MPTP+CE 5mg/kg
MPTP+CE 10mg/kg
MPTP+CE 20mg/kg
L
L
L
R
R
R

## Slide 10
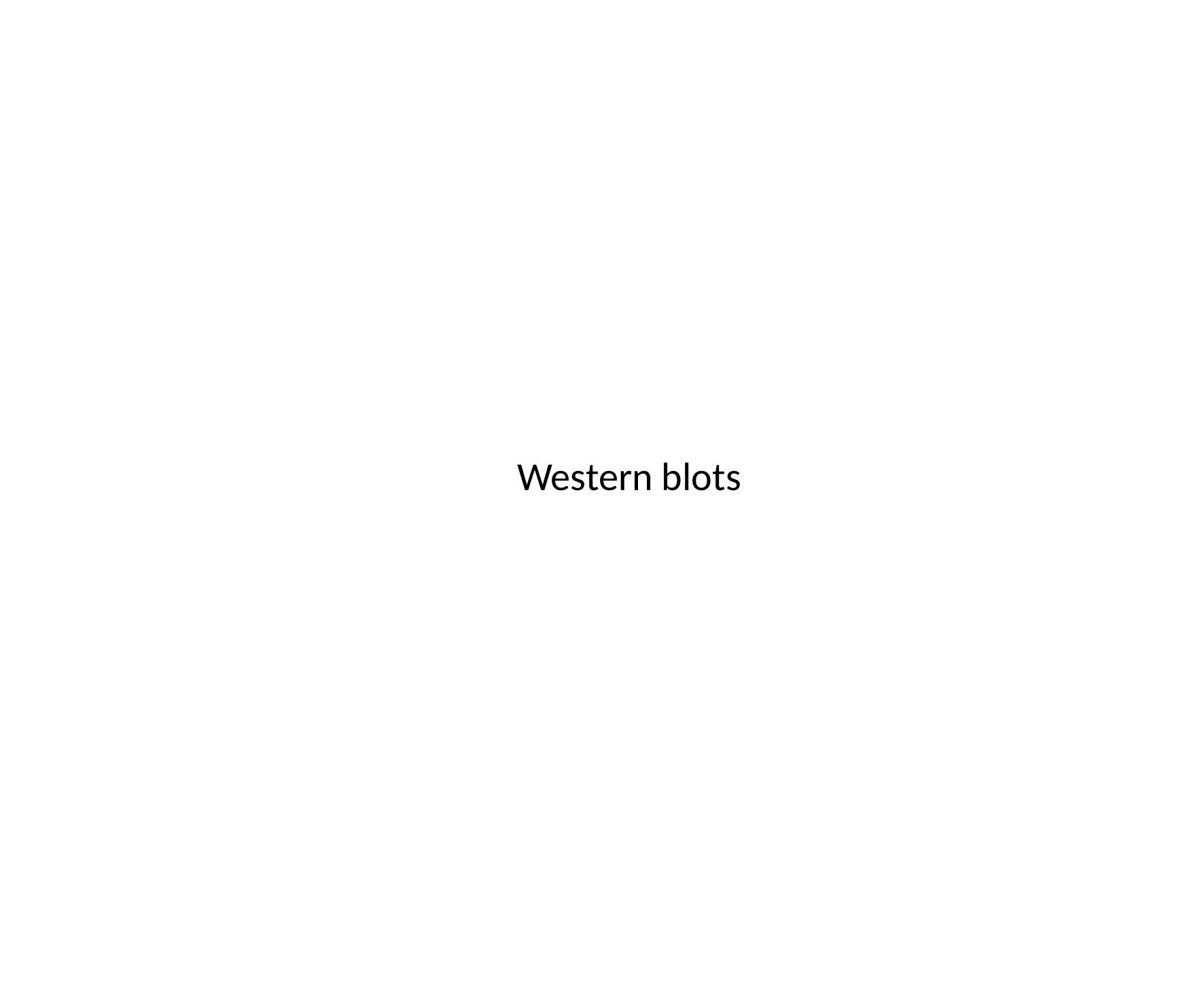

Western blots

## Slide 11
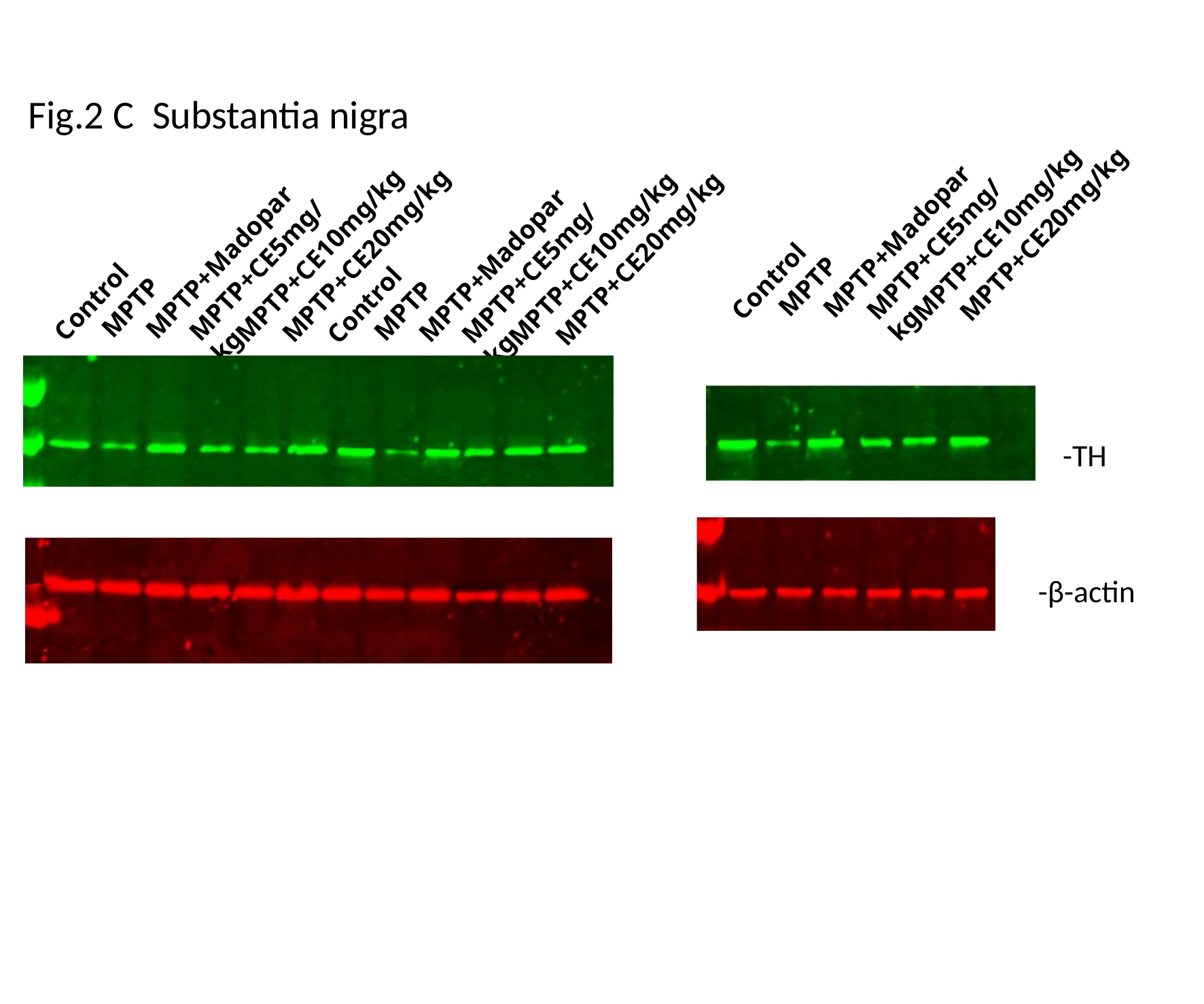

Fig.2 C Substantia nigra
MPTP+CE10mg/kg
MPTP+CE20mg/kg
MPTP+Madopar
MPTP+CE5mg/kg
MPTP
Control
MPTP+CE10mg/kg
MPTP+CE20mg/kg
MPTP+Madopar
MPTP+CE5mg/kg
MPTP
Control
MPTP+CE10mg/kg
MPTP+CE20mg/kg
MPTP+Madopar
MPTP+CE5mg/kg
MPTP
Control
-TH
-β-actin

## Slide 12
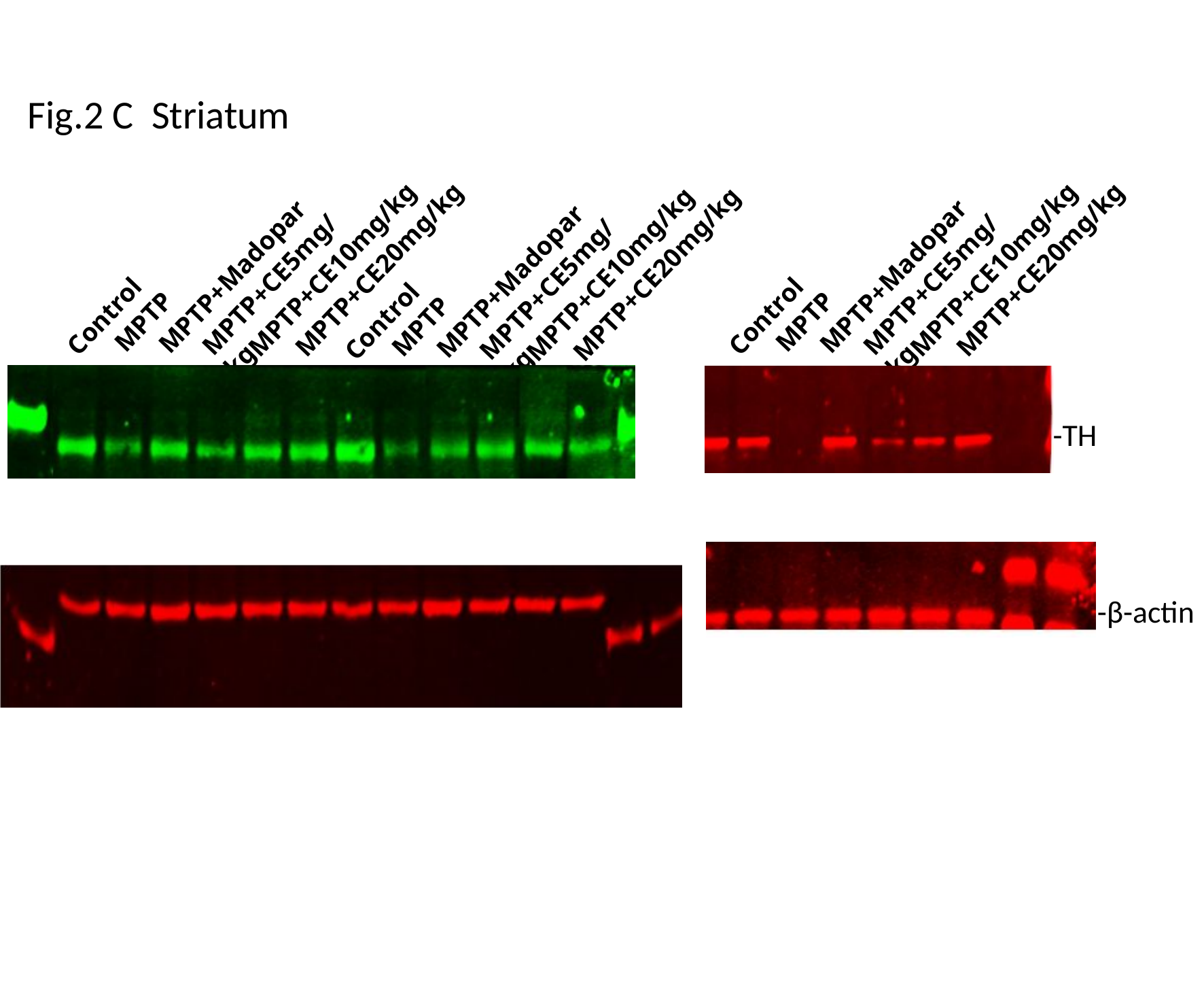

Fig.2 C Striatum
MPTP+CE10mg/kg
MPTP+CE20mg/kg
MPTP+Madopar
MPTP+CE5mg/kg
MPTP
Control
MPTP+CE10mg/kg
MPTP+CE20mg/kg
MPTP+Madopar
MPTP+CE5mg/kg
MPTP
Control
MPTP+CE10mg/kg
MPTP+CE20mg/kg
MPTP+Madopar
MPTP+CE5mg/kg
MPTP
Control
-TH
-β-actin

## Slide 13
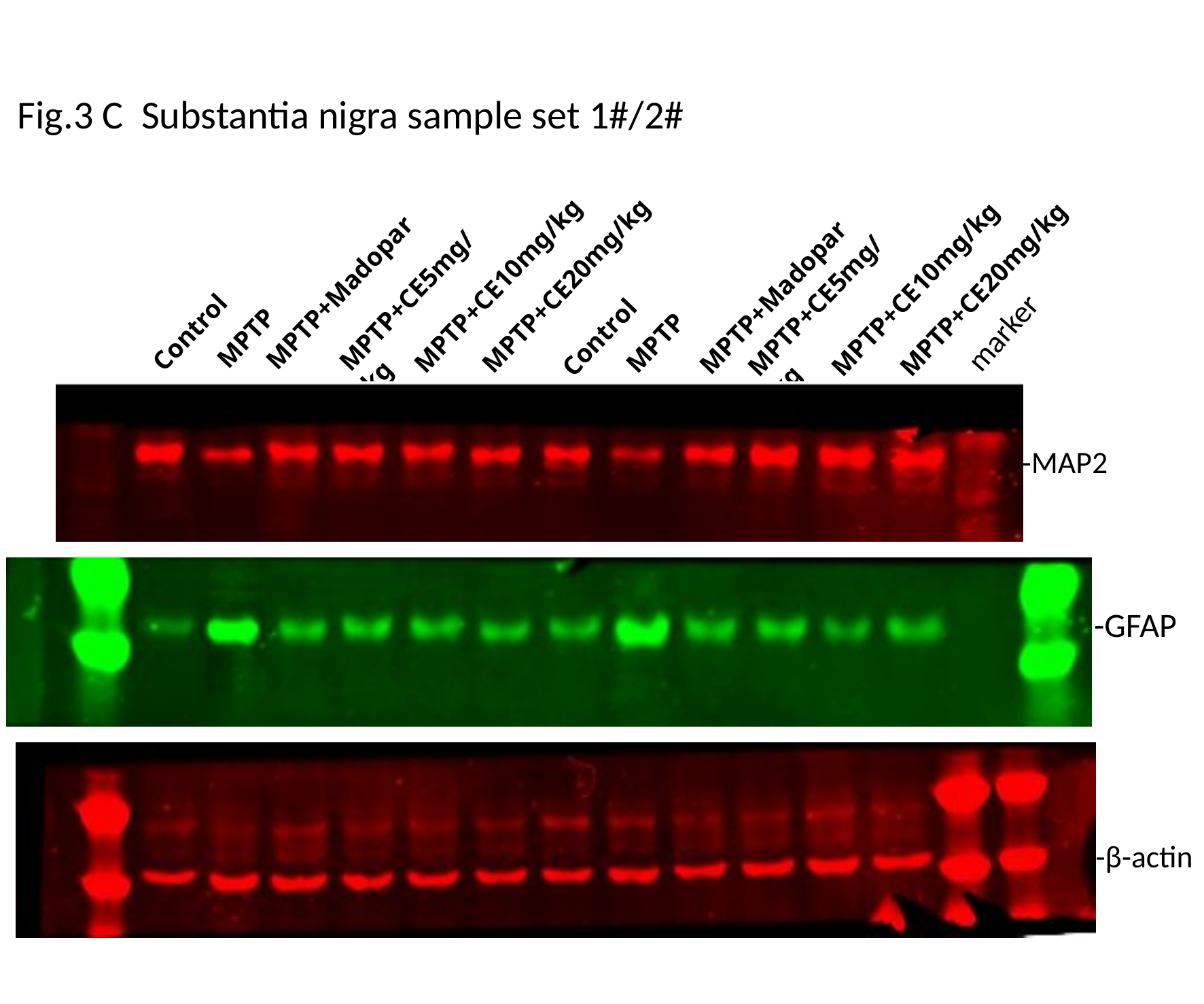

Fig.3 C Substantia nigra sample set 1#/2#
MPTP+CE10mg/kg
MPTP+CE20mg/kg
MPTP+Madopar
MPTP+CE5mg/kg
MPTP
Control
MPTP+CE10mg/kg
MPTP+CE20mg/kg
MPTP+Madopar
MPTP+CE5mg/kg
MPTP
Control
marker
-MAP2
-GFAP
-β-actin

## Slide 14
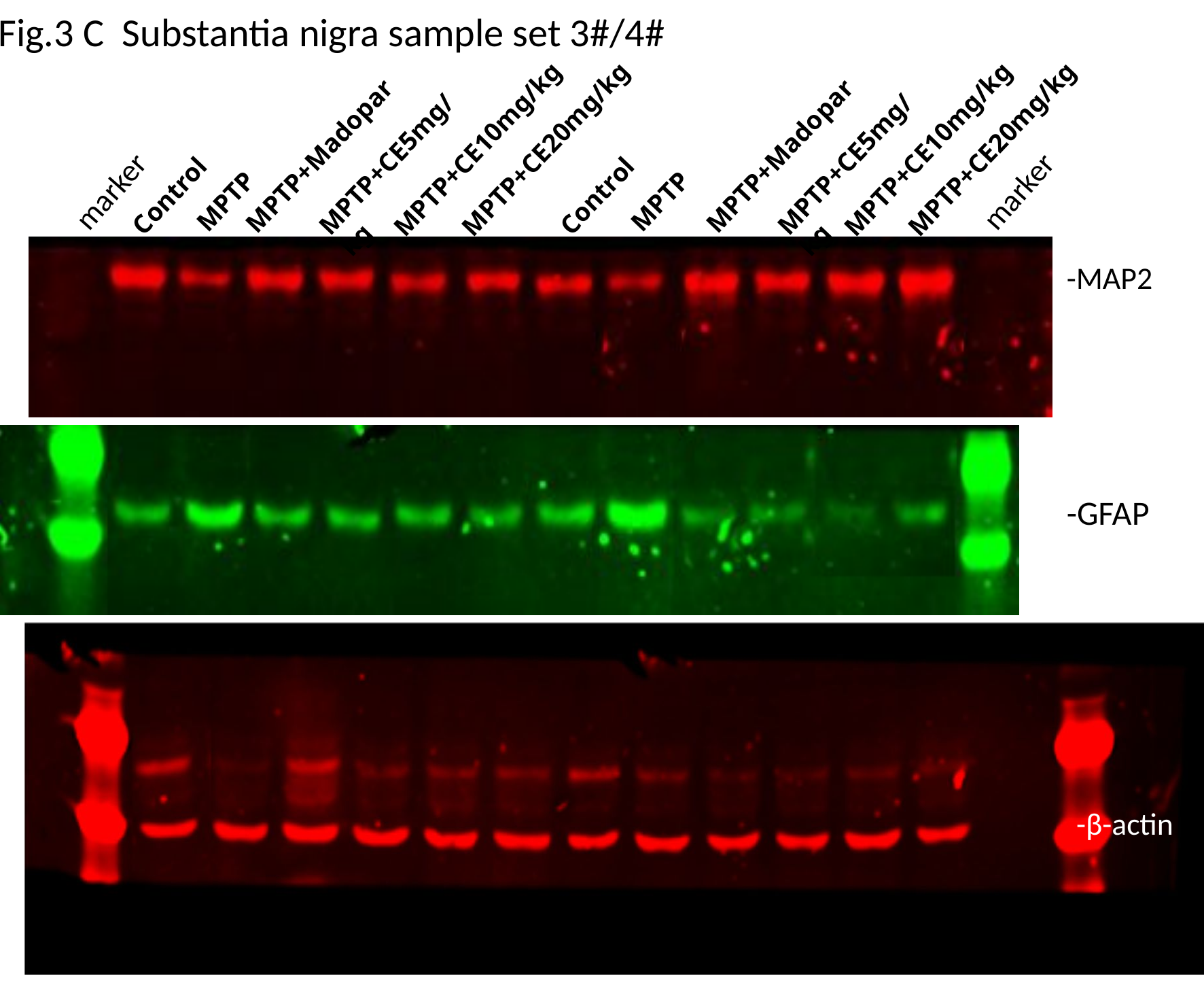

MPTP+CE10mg/kg
MPTP+CE20mg/kg
MPTP+Madopar
MPTP+CE5mg/kg
MPTP
Control
MPTP+CE10mg/kg
MPTP+CE20mg/kg
MPTP+Madopar
MPTP+CE5mg/kg
MPTP
Control
Fig.3 C Substantia nigra sample set 3#/4#
marker
marker
-MAP2
-GFAP
-β-actin

## Slide 15
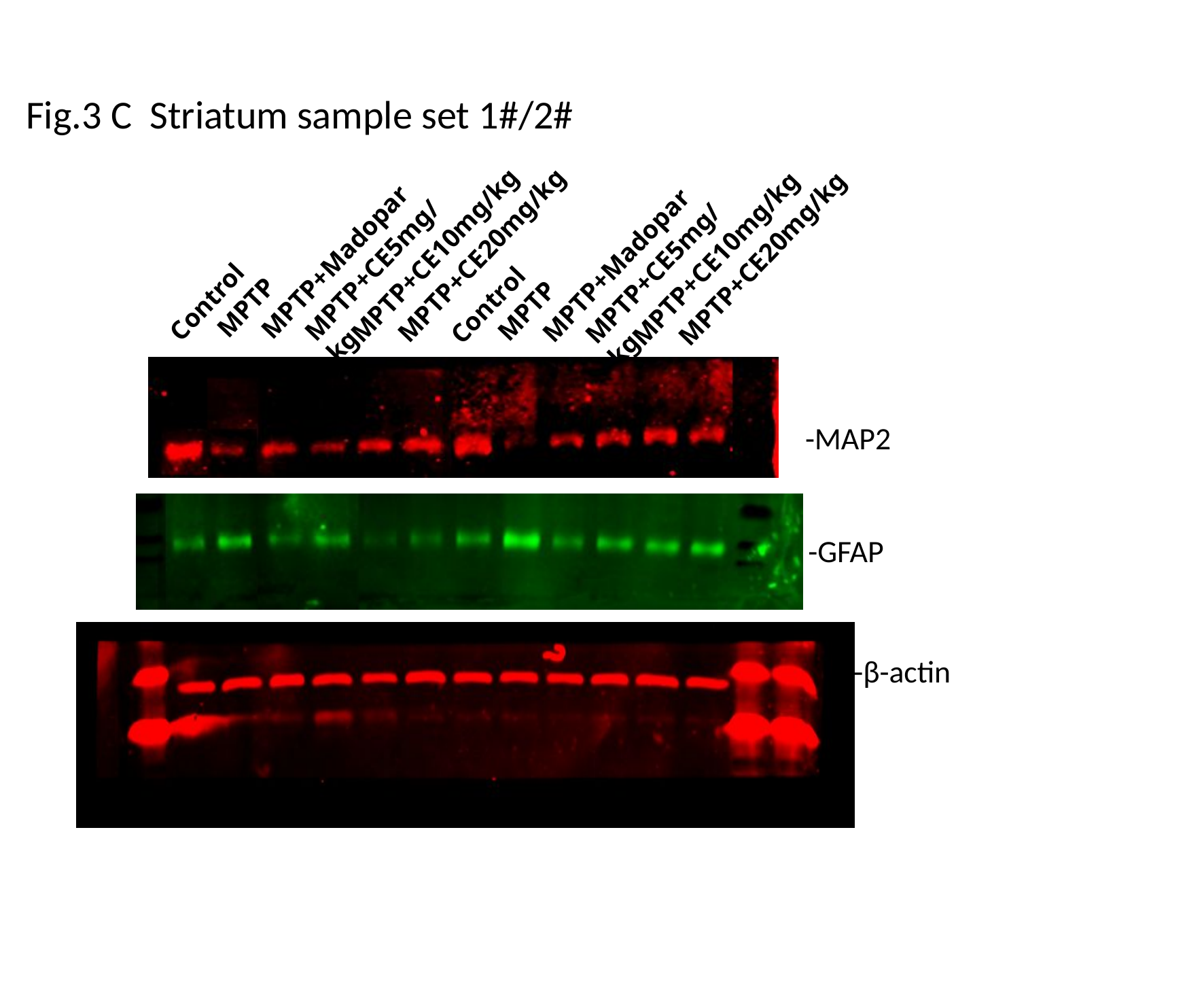

Fig.3 C Striatum sample set 1#/2#
MPTP+CE10mg/kg
MPTP+CE20mg/kg
MPTP+Madopar
MPTP+CE5mg/kg
MPTP
Control
MPTP+CE10mg/kg
MPTP+CE20mg/kg
MPTP+Madopar
MPTP+CE5mg/kg
MPTP
Control
-MAP2
-GFAP
-β-actin

## Slide 16
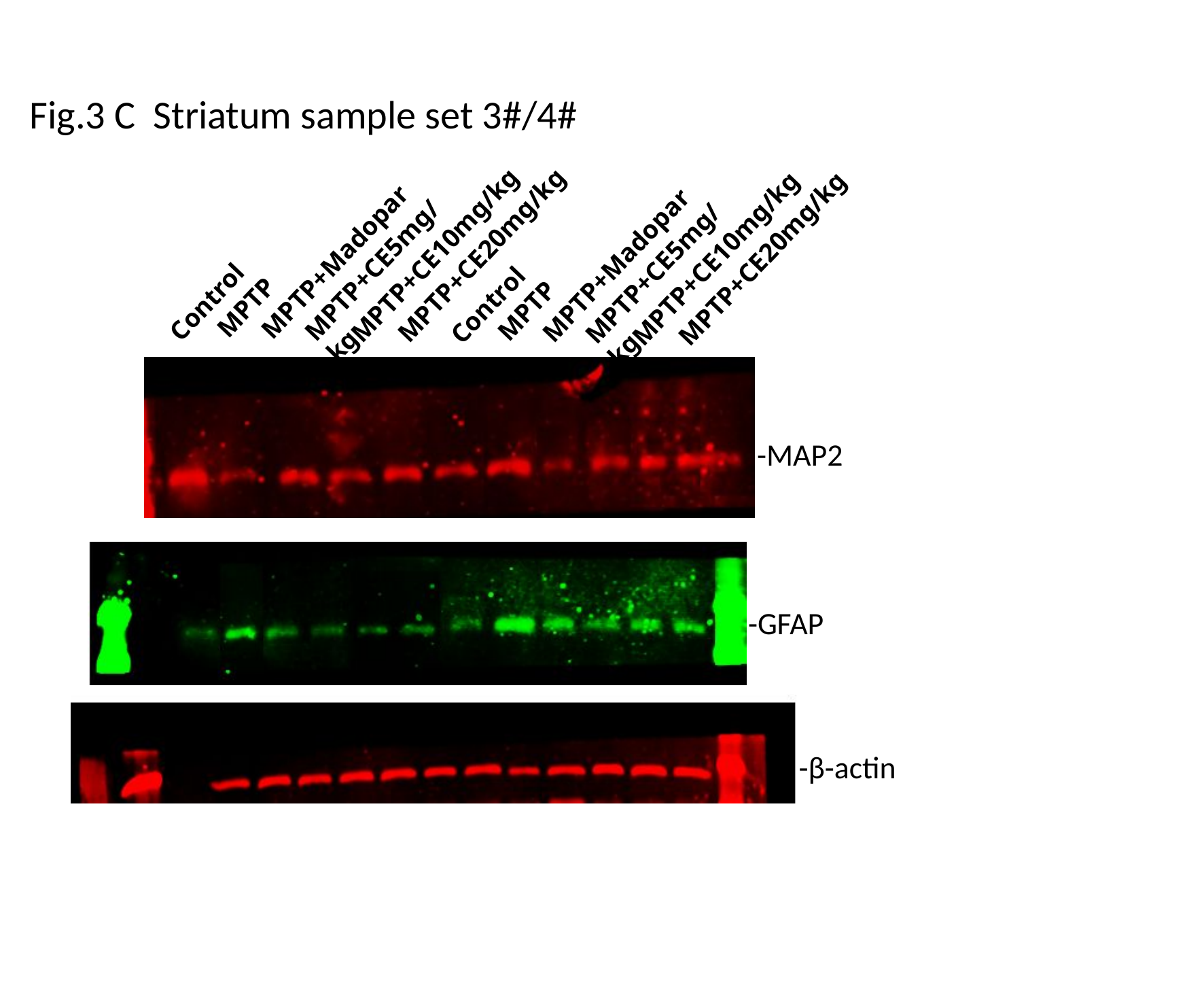

Fig.3 C Striatum sample set 3#/4#
MPTP+CE10mg/kg
MPTP+CE20mg/kg
MPTP+Madopar
MPTP+CE5mg/kg
MPTP
Control
MPTP+CE10mg/kg
MPTP+CE20mg/kg
MPTP+Madopar
MPTP+CE5mg/kg
MPTP
Control
-MAP2
-GFAP
-β-actin

## Slide 17
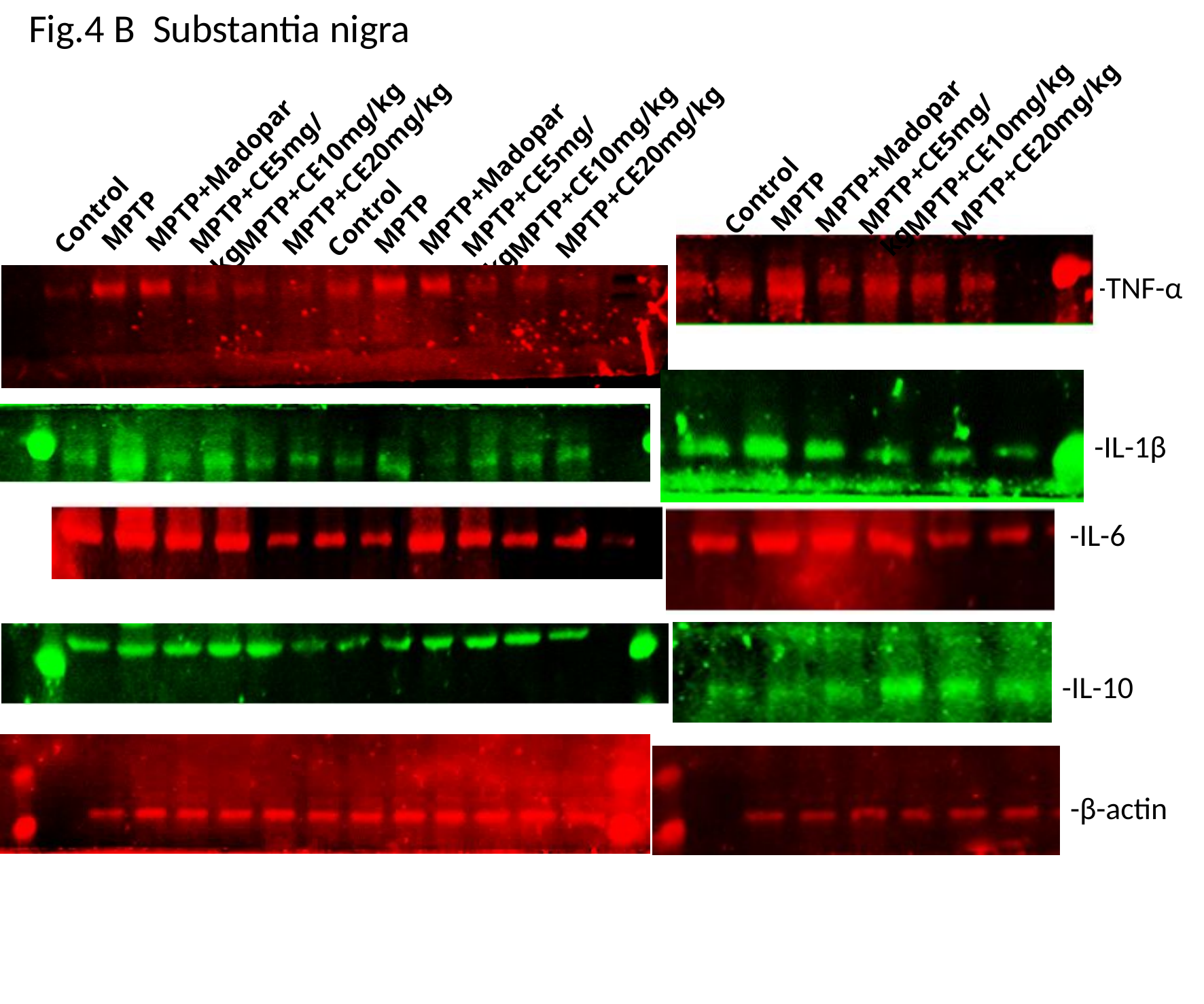

Fig.4 B Substantia nigra
MPTP+CE10mg/kg
MPTP+CE20mg/kg
MPTP+Madopar
MPTP+CE5mg/kg
MPTP
Control
MPTP+CE10mg/kg
MPTP+CE20mg/kg
MPTP+Madopar
MPTP+CE5mg/kg
MPTP
Control
MPTP+CE10mg/kg
MPTP+CE20mg/kg
MPTP+Madopar
MPTP+CE5mg/kg
MPTP
Control
-TNF-α
-IL-1β
-IL-6
-IL-10
-β-actin

## Slide 18
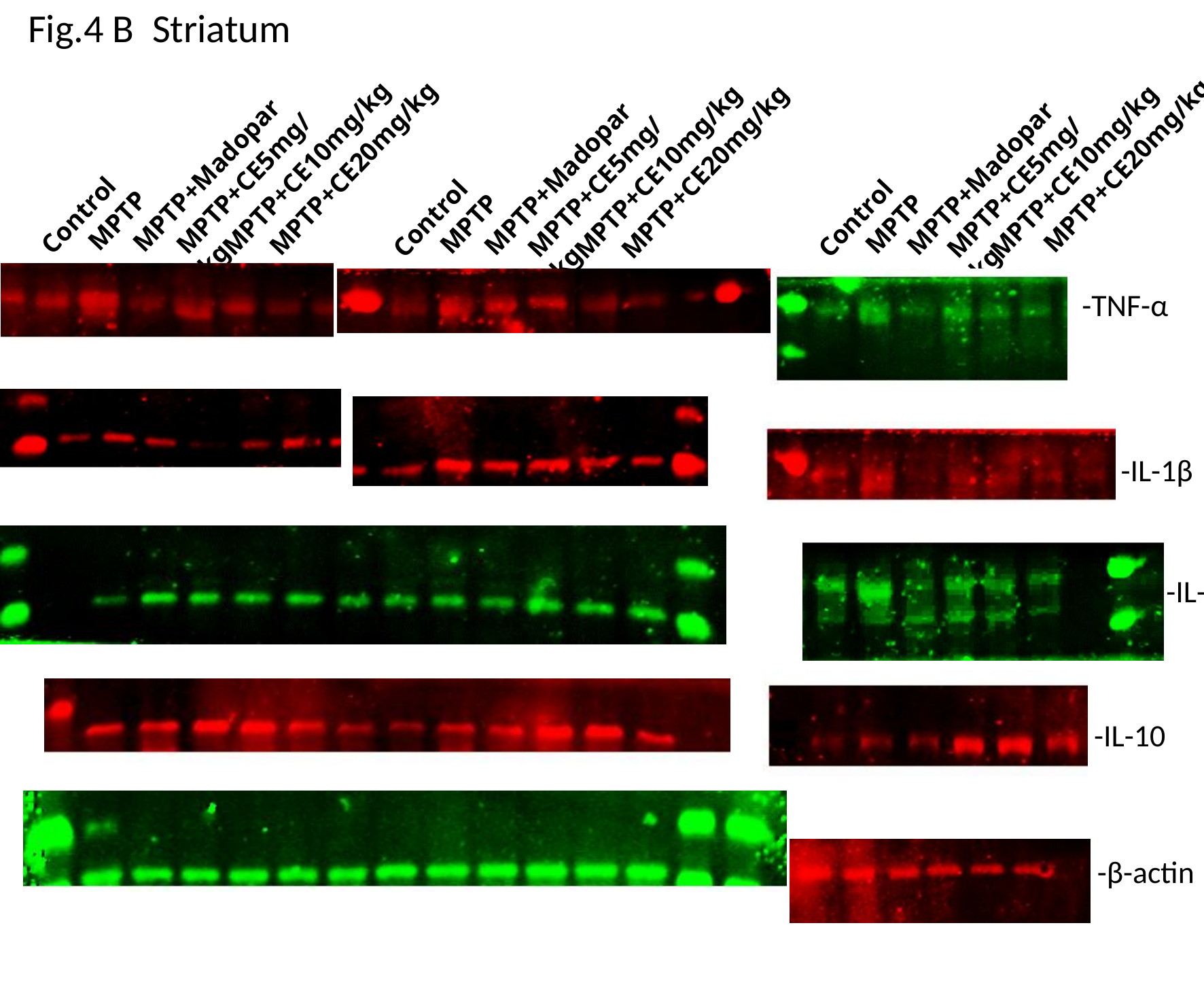

Fig.4 B Striatum
MPTP+CE10mg/kg
MPTP+CE20mg/kg
MPTP+Madopar
MPTP+CE5mg/kg
MPTP
Control
MPTP+CE10mg/kg
MPTP+CE20mg/kg
MPTP+Madopar
MPTP+CE5mg/kg
MPTP
Control
MPTP+CE10mg/kg
MPTP+CE20mg/kg
MPTP+Madopar
MPTP+CE5mg/kg
MPTP
Control
-TNF-α
-IL-1β
-IL-6
-IL-10
-β-actin

## Slide 19
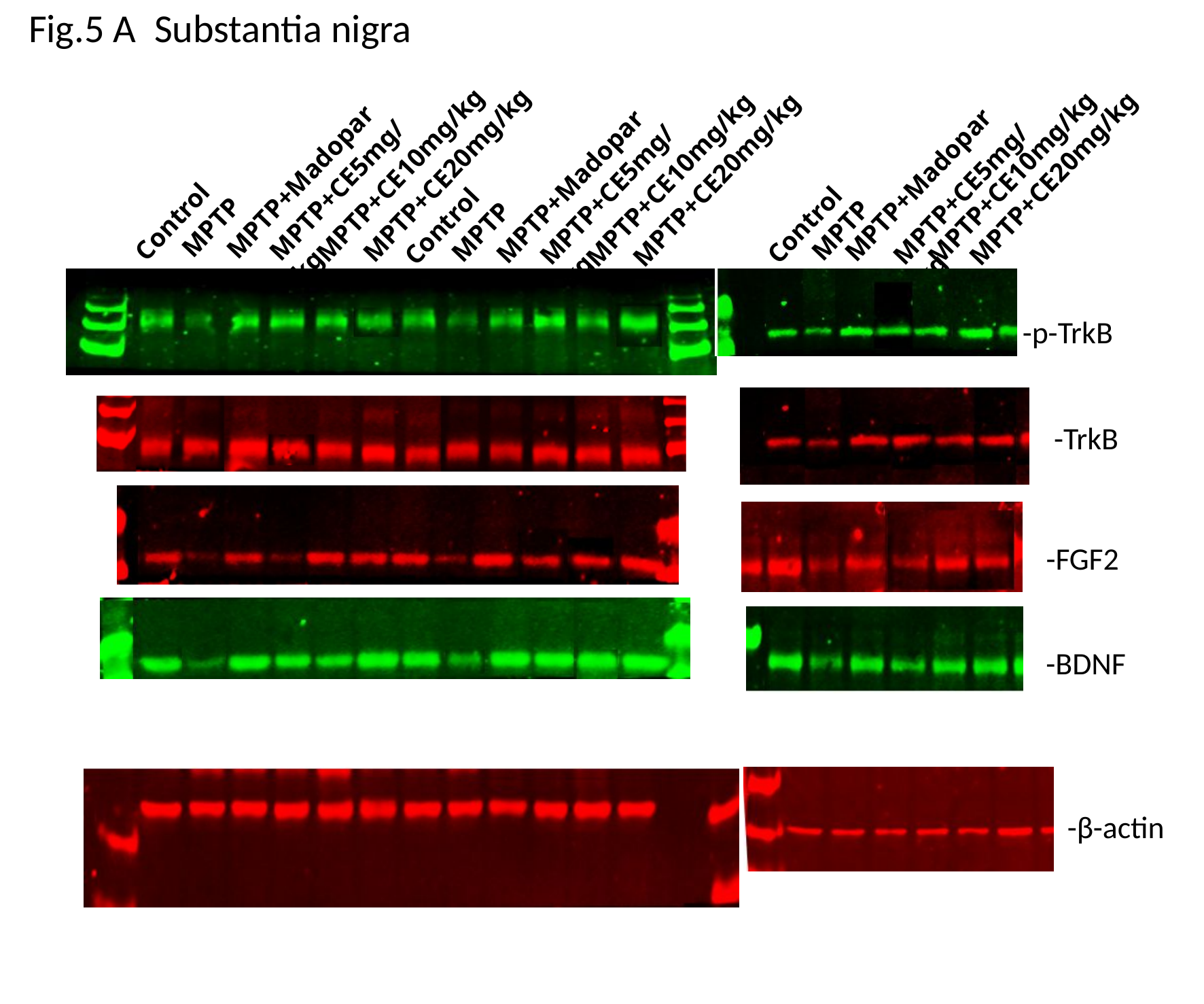

Fig.5 A Substantia nigra
MPTP+CE10mg/kg
MPTP+CE20mg/kg
MPTP+Madopar
MPTP+CE5mg/kg
MPTP
Control
MPTP+CE10mg/kg
MPTP+CE20mg/kg
MPTP+Madopar
MPTP+CE5mg/kg
MPTP
Control
MPTP+CE10mg/kg
MPTP+CE20mg/kg
MPTP+Madopar
MPTP+CE5mg/kg
MPTP
Control
-p-TrkB
-TrkB
-FGF2
-BDNF
-β-actin

## Slide 20
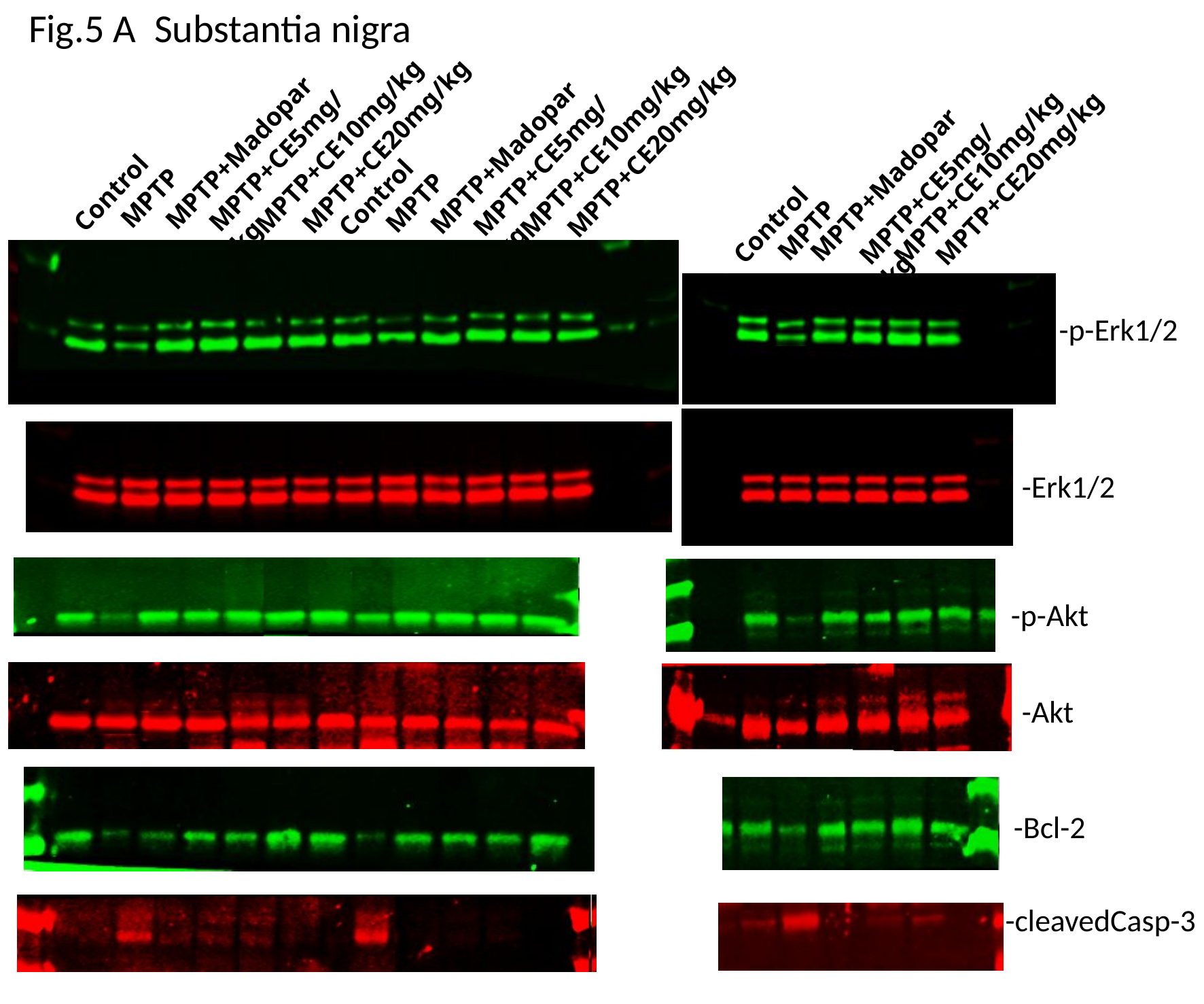

MPTP+CE10mg/kg
MPTP+CE20mg/kg
MPTP+Madopar
MPTP+CE5mg/kg
MPTP
Control
Fig.5 A Substantia nigra
MPTP+CE10mg/kg
MPTP+CE20mg/kg
MPTP+Madopar
MPTP+CE5mg/kg
MPTP
Control
MPTP+CE10mg/kg
MPTP+CE20mg/kg
MPTP+Madopar
MPTP+CE5mg/kg
MPTP
Control
-p-Erk1/2
-Erk1/2
-p-Akt
-Akt
-Bcl-2
-cleavedCasp-3

## Slide 21
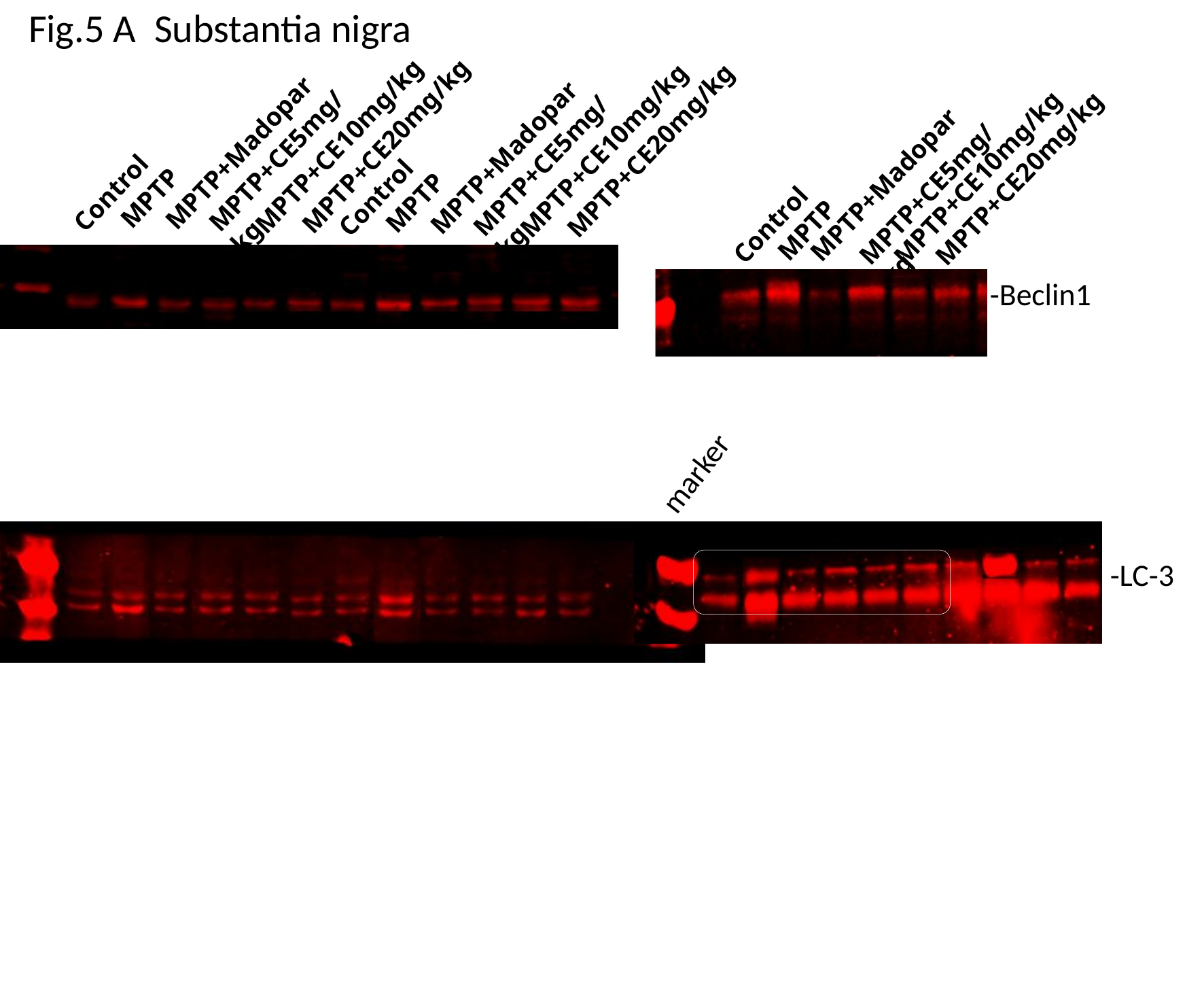

MPTP+CE10mg/kg
MPTP+CE20mg/kg
MPTP+Madopar
MPTP+CE5mg/kg
MPTP
Control
Fig.5 A Substantia nigra
MPTP+CE10mg/kg
MPTP+CE20mg/kg
MPTP+Madopar
MPTP+CE5mg/kg
MPTP
Control
MPTP+CE10mg/kg
MPTP+CE20mg/kg
MPTP+Madopar
MPTP+CE5mg/kg
MPTP
Control
-Beclin1
marker
-LC-3

## Slide 22
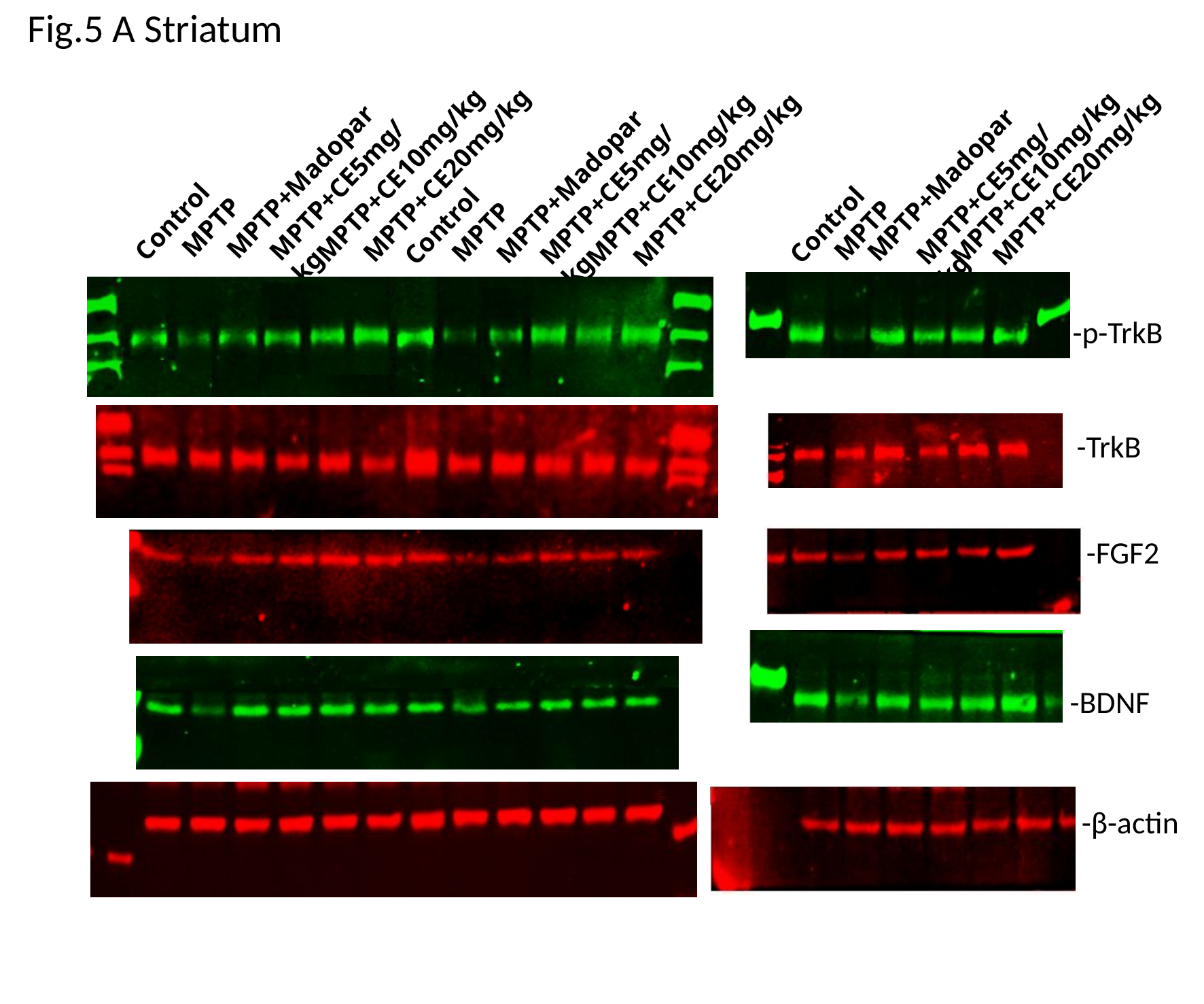

Fig.5 A Striatum
MPTP+CE10mg/kg
MPTP+CE20mg/kg
MPTP+Madopar
MPTP+CE5mg/kg
MPTP
Control
MPTP+CE10mg/kg
MPTP+CE20mg/kg
MPTP+Madopar
MPTP+CE5mg/kg
MPTP
Control
MPTP+CE10mg/kg
MPTP+CE20mg/kg
MPTP+Madopar
MPTP+CE5mg/kg
MPTP
Control
-p-TrkB
-TrkB
-FGF2
-BDNF
-β-actin

## Slide 23
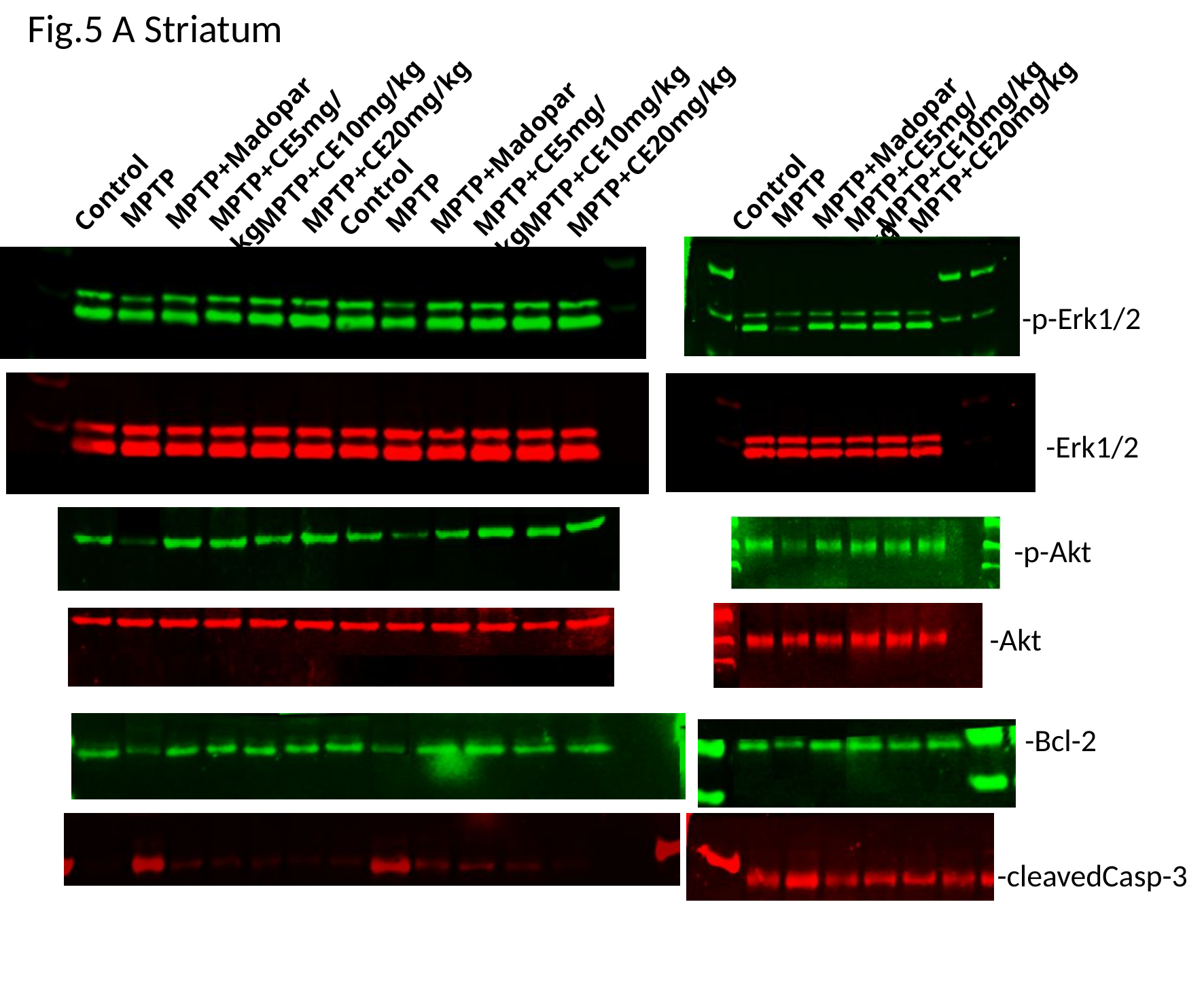

MPTP+CE10mg/kg
MPTP+CE20mg/kg
MPTP+Madopar
MPTP+CE5mg/kg
MPTP
Control
MPTP+CE10mg/kg
MPTP+CE20mg/kg
MPTP+Madopar
MPTP+CE5mg/kg
MPTP
Control
Fig.5 A Striatum
MPTP+CE10mg/kg
MPTP+CE20mg/kg
MPTP+Madopar
MPTP+CE5mg/kg
MPTP
Control
-p-Erk1/2
-Erk1/2
-p-Akt
-Akt
-Bcl-2
-cleavedCasp-3

## Slide 24
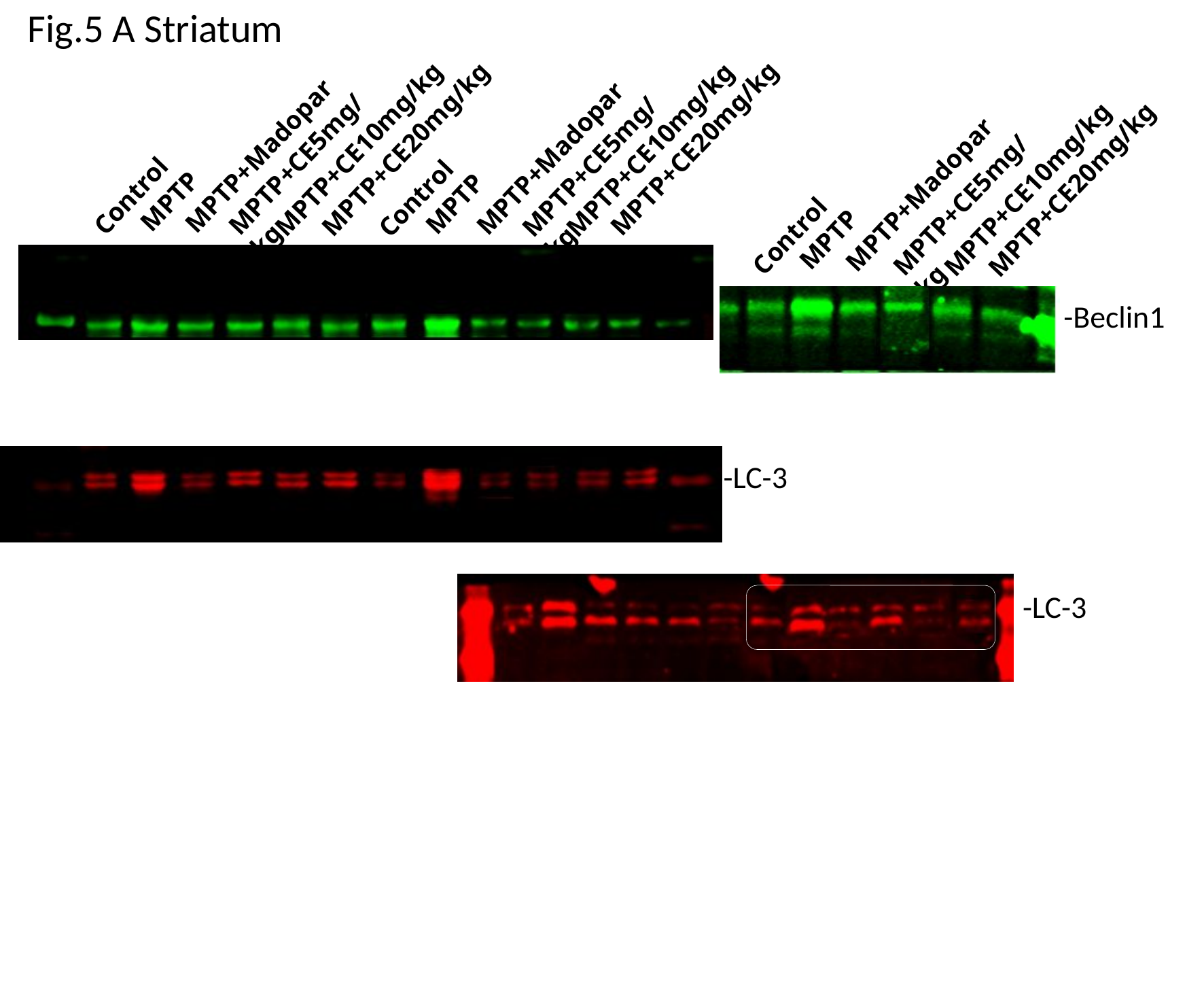

MPTP+CE10mg/kg
MPTP+CE20mg/kg
MPTP+Madopar
MPTP+CE5mg/kg
MPTP
Control
Fig.5 A Striatum
MPTP+CE10mg/kg
MPTP+CE20mg/kg
MPTP+Madopar
MPTP+CE5mg/kg
MPTP
Control
MPTP+CE10mg/kg
MPTP+CE20mg/kg
MPTP+Madopar
MPTP+CE5mg/kg
MPTP
Control
-Beclin1
-LC-3
-LC-3

## Slide 25
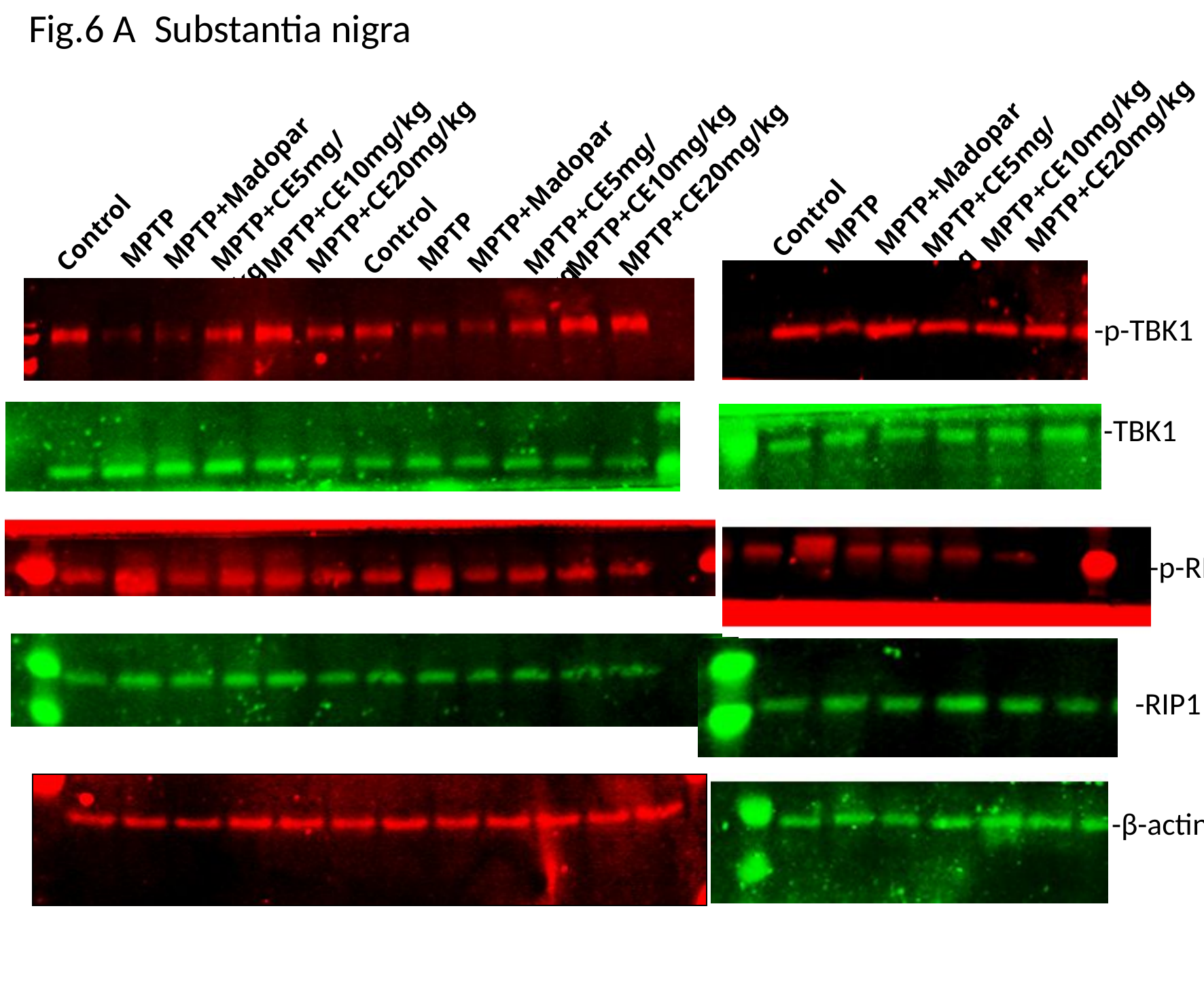

Fig.6 A Substantia nigra
MPTP+CE10mg/kg
MPTP+CE20mg/kg
MPTP+Madopar
MPTP+CE5mg/kg
MPTP
Control
MPTP+CE10mg/kg
MPTP+CE20mg/kg
MPTP+Madopar
MPTP+CE5mg/kg
MPTP
Control
MPTP+CE10mg/kg
MPTP+CE20mg/kg
MPTP+Madopar
MPTP+CE5mg/kg
MPTP
Control
-p-TBK1
-TBK1
-p-RIP1
-RIP1
-β-actin

## Slide 26
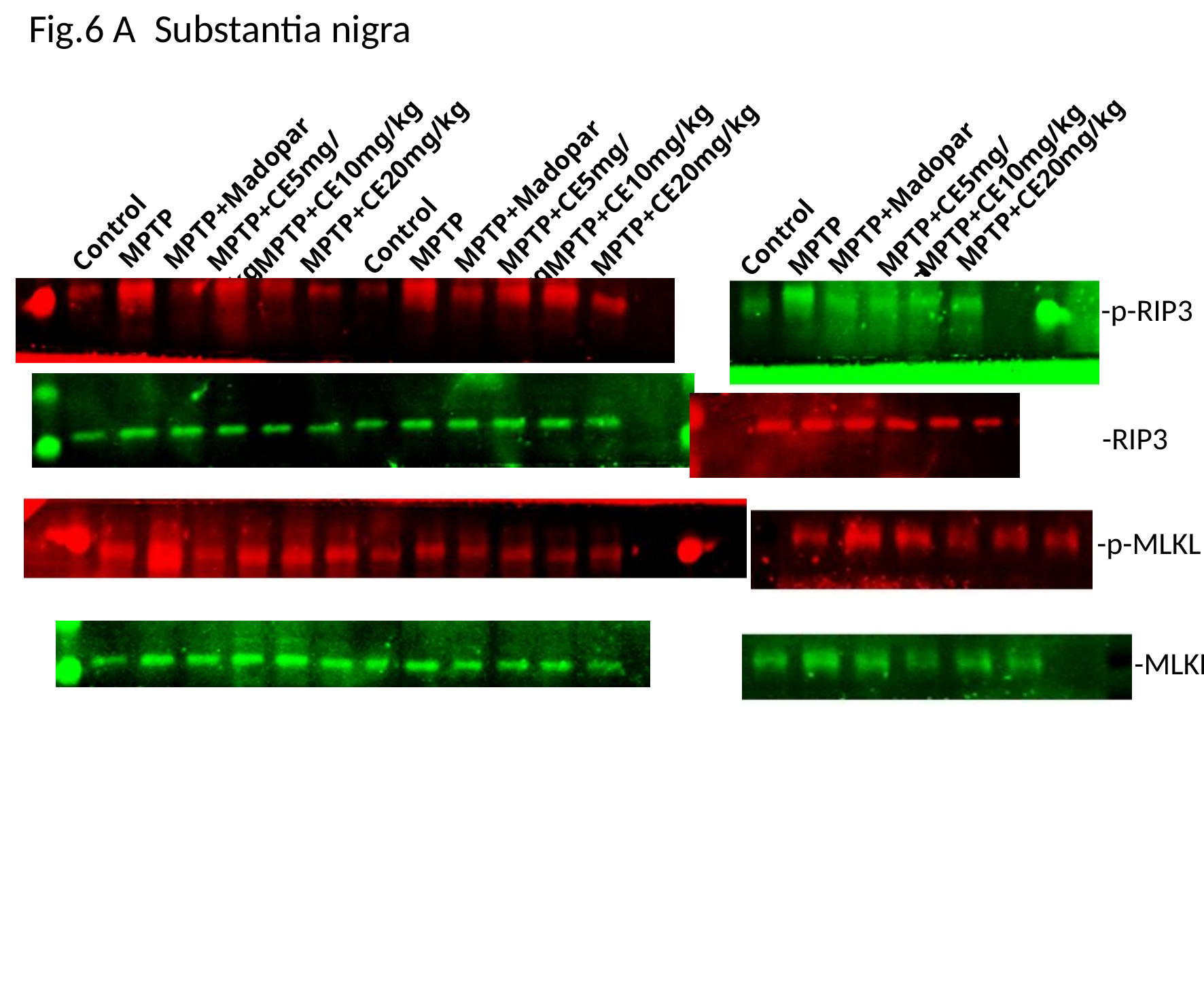

Fig.6 A Substantia nigra
MPTP+CE10mg/kg
MPTP+CE20mg/kg
MPTP+Madopar
MPTP+CE5mg/kg
MPTP
Control
MPTP+CE10mg/kg
MPTP+CE20mg/kg
MPTP+Madopar
MPTP+CE5mg/kg
MPTP
Control
MPTP+CE10mg/kg
MPTP+CE20mg/kg
MPTP+Madopar
MPTP+CE5mg/kg
MPTP
Control
-p-RIP3
-RIP3
-p-MLKL
-MLKL

## Slide 27
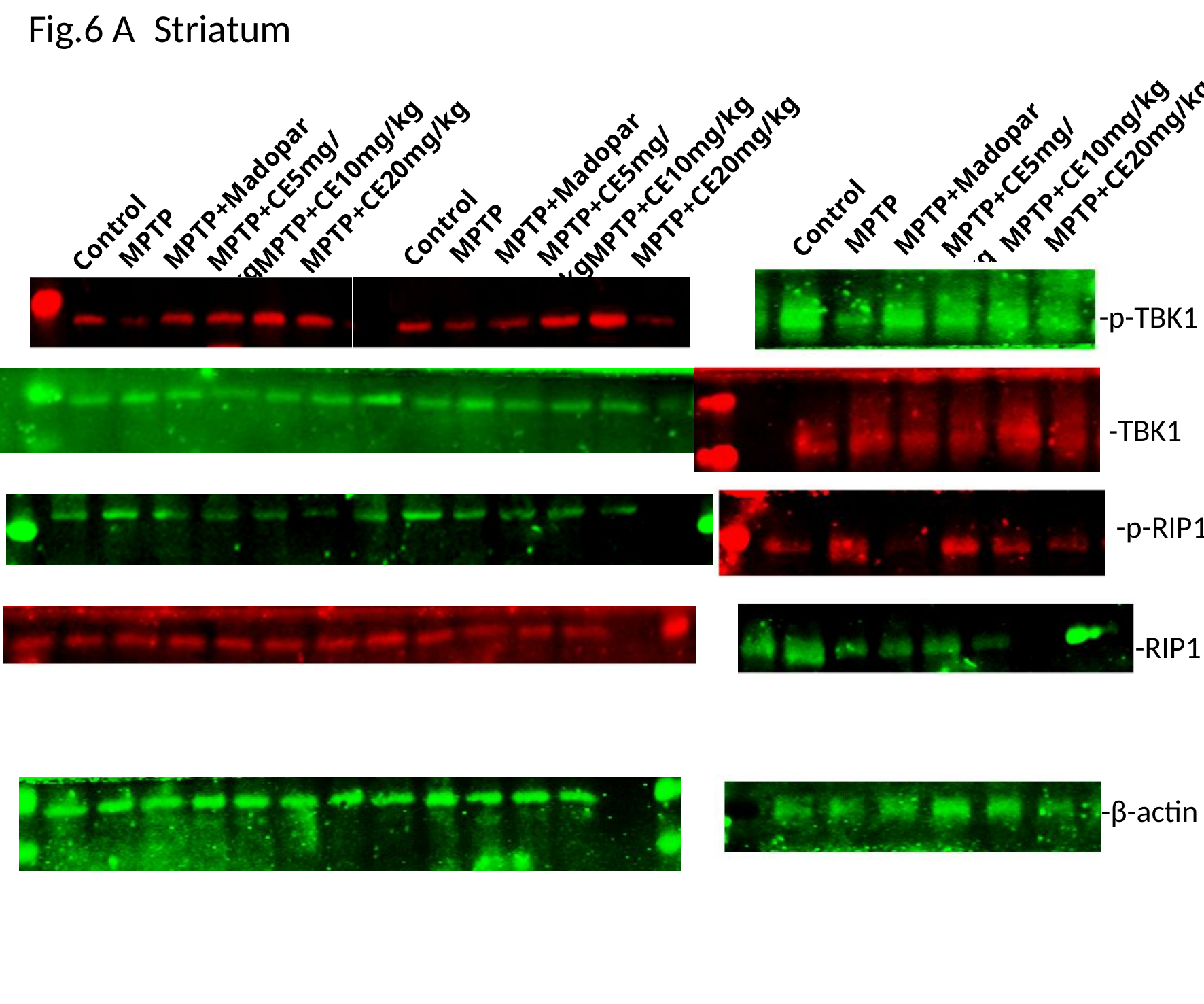

Fig.6 A Striatum
MPTP+CE10mg/kg
MPTP+CE20mg/kg
MPTP+Madopar
MPTP+CE5mg/kg
MPTP
Control
MPTP+CE10mg/kg
MPTP+CE20mg/kg
MPTP+Madopar
MPTP+CE5mg/kg
MPTP
Control
MPTP+CE10mg/kg
MPTP+CE20mg/kg
MPTP+Madopar
MPTP+CE5mg/kg
MPTP
Control
-p-TBK1
-TBK1
-p-RIP1
-RIP1
-β-actin

## Slide 28
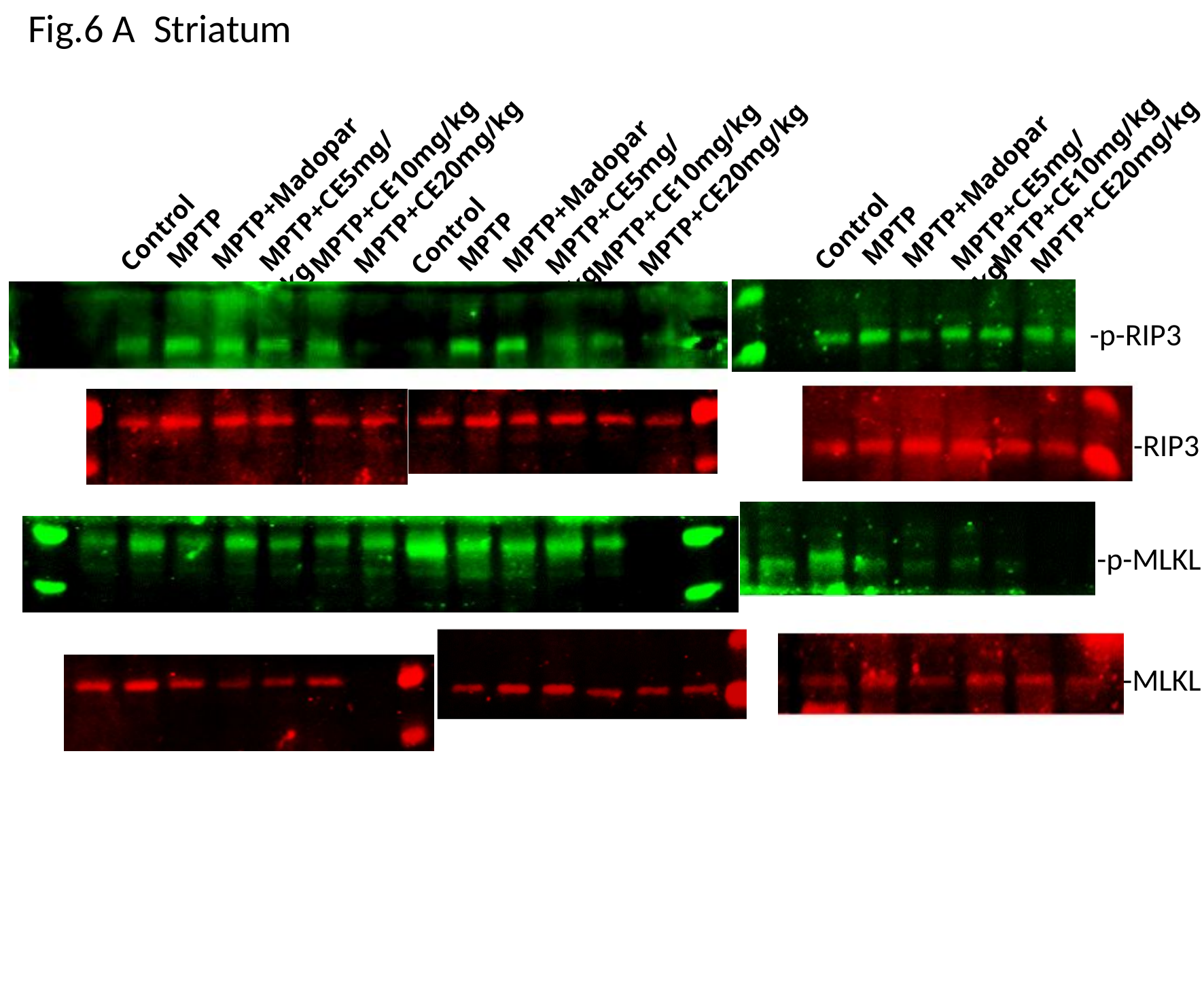

Fig.6 A Striatum
MPTP+CE10mg/kg
MPTP+CE20mg/kg
MPTP+Madopar
MPTP+CE5mg/kg
MPTP
Control
MPTP+CE10mg/kg
MPTP+CE20mg/kg
MPTP+Madopar
MPTP+CE5mg/kg
MPTP
Control
MPTP+CE10mg/kg
MPTP+CE20mg/kg
MPTP+Madopar
MPTP+CE5mg/kg
MPTP
Control
-p-RIP3
-RIP3
-p-MLKL
-MLKL
